# Polychromatic full-polarization control in mid-infrared light

**DOI:** 10.1038/s41377-023-01140-3

**Published:** 2023-05-04

**Authors:** Jin Chen, Feilong Yu, Xingsi Liu, Yanjun Bao, Rongsheng Chen, Zengyue Zhao, Jiuxu Wang, Xiuxia Wang, Wen Liu, Yuzhi Shi, Cheng-Wei Qiu, Xiaoshuang Chen, Wei Lu, Guanhai Li

**Affiliations:** 1 State Key Laboratory of Infrared Physics, Shanghai Institute of Technical Physics, Chinese Academy of Sciences, 500 Yu Tian Road, Shanghai, 200083 China; 2 Hangzhou Institute for Advanced Study, University of Chinese Academy of Sciences, No. 1 SubLane Xiangshan, Hangzhou, 310024 China; 3 Shanghai Research Center for Quantum Sciences, 99 Xiupu Road, Shanghai, 201315 China; 4 University of Chinese Academy of Science, No. 19 Yuquan Road, Beijing, 100049 China; 5 Department of Electrical and Computer Engineering, National University of Singapore, 4 Engineering Drive 3, Singapore, 117583 Singapore; 6 Institute of Nanophotonics, Jinan University, Guangzhou, 511443 China; 7 Center for Micro-and Nanoscale Research and Fabrication, University of Science and Technology of China, Hefei, 230026 China; 8 Institute of Precision Optical Engineering, School of Physics Science and Engineering, Tongji University, Shanghai, 200092 China; 9 National University of Singapore Suzhou Research Institute, No. 377 Linquan Street, Suzhou, Jiangsu 215123 China

**Keywords:** Mid-infrared photonics, Metamaterials, Imaging and sensing, Micro-optics

## Abstract

Objects with different shapes, materials and temperatures can emit distinct polarizations and spectral information in mid-infrared band, which provides a unique signature in the transparent window for object identification. However, the crosstalk among various polarization and wavelength channels prevents from accurate mid-infrared detections at high signal-to-noise ratio. Here, we report full-polarization metasurfaces to break the inherent eigen-polarization constraint over the wavelengths in mid-infrared. This recipe enables to select arbitrary orthogonal polarization basis at individual wavelength independently, therefore alleviating the crosstalk and efficiency degradation. A six-channel all-silicon metasurface is specifically presented to project focused mid-infrared light to distinct positions at three wavelengths, each with a pair of arbitrarily chosen orthogonal polarizations. An isolation ratio of 117 between neighboring polarization channels is experimentally recorded, exhibiting detection sensitivity one order of magnitude higher than existing infrared detectors. Remarkably, the high aspect ratio ~30 of our meta-structures manufactured by deep silicon etching technology at temperature −150 °C guarantees the large and precise phase dispersion control over a broadband from 3 to 4.5 μm. We believe our results would benefit the noise-immune mid-infrared detections in remote sensing and space-to-ground communications.

## Abstract (web summary)

Dispersive eigen-polarization engineering enabled polychromatic full-polarization control in mid-infrared.

## Graphical abstract

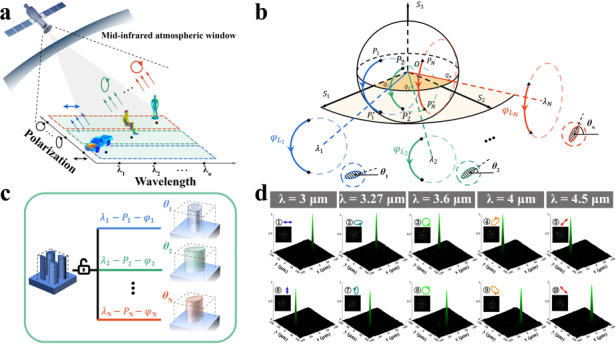

## Introduction

Mid-wavelength infrared (MWIR) as one of the most important transparent atmosphere windows is less sensitive to the interference from the background emission of the sun, providing a high-transmission zone for the finger-print spectra of various materials, and enabling the communication channels between space and ground. As fundamental characteristics of photon, wavelength, polarization and their measurements are of great interest in almost all areas of science and in remote sensing technology. Traditionally, infrared detection techniques are solely detecting the intensity, which can be easily overwhelmed by the path loss, weather variation, as well as the atmosphere turbulence. In comparison, the information from wavelength and polarization dimensions can reveal distinct features that are intuitively “invisible”^1–3^. Owing to the merits of robustly identifying true targets from the false and improving the contrast of captured images, it is imperative to develop methods to detect photon dimensions other than intensity, such as wavelength (*λ*) and polarization (*P*).

**Fig. 1:**
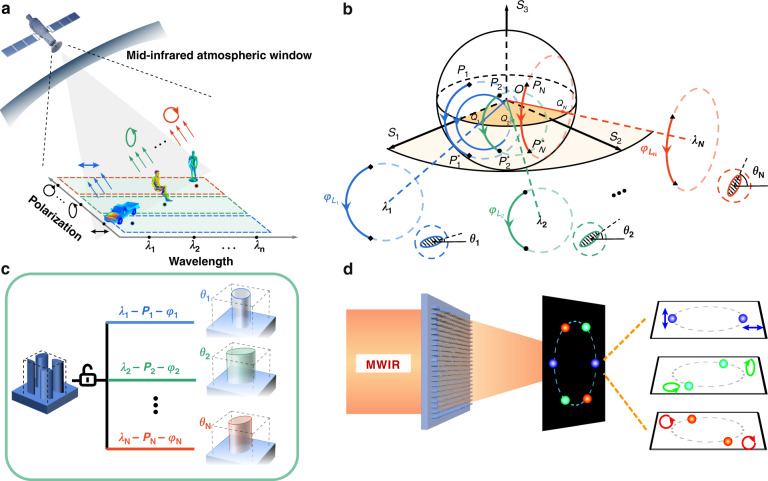
The principle of the polychromatic full-polarization control. **a** A typical scenario in which complex targets with different spectral and polarization information in low-illumination background need to be distinguished by airborne or spaceborne payloads. **b** In traditional works based on geometric phase control, different rotation angles *θ* of the metaatoms represent different eigen-polarization states (EPS). In the Poincaré sphere, EPS is denoted by the precession axis *OQ*. The arcs in Poincaré sphere indicate polarization conversion paths and their lengths *L* determine the phase shifts *φ*_*L*_. **c** Schematic of a wavelength-decoupled coherent pixel. The architecture consists of four ordinary metaatoms with adjustable dimensions and orientations. With this configuration, untraditional phase responses at different wavelengths can be simultaneously and independently harnessed, thus enabling desired functionalities on any designated polarization state channels. The common geometric parameters of the metaatoms are: height 6 μm, period 1.55 μm. The supercell period is 3.1 μm. **d** Schematic of a representative metadevice which achromatically focuses the incident light at three discrete wavelengths (3, 3.6, and 4.5 μm)—each assigned with a pair of arbitrarily chosen orthogonal polarizations—onto two opposite vertices of the hexagon

A typical scenario for the onboard payloads which are carried in satellites or airplanes to capture fine features of targets on the ground [car (*λ*_1_, *P*_1_), man (*λ*_2_, *P*_2_), and model (*λ*_3_, *P*_3_)] in a low-illumination background is shown in Fig. 1a. In order to obtain the information from different wavelengths and polarizations, a series of efforts have been paid. Since the undifferentiated integral detection of the spectrum and polarization reported in 1917^4,5^, complex optical systems with cascaded elements have been explored to separately acquire spectrum or polarization information through rotating the discrete filters/polarizers or segmenting the detecting focal plane arrays from 1970s^6–11^. These configurations suffer from bulky and redundant volume, low collection efficiency of photons, etc. More importantly, they were realized at the expense of decreasing spatial or temporal resolutions.

Metasurfaces that enable versatile nanoscale light control in manipulating multiple photon dimensions, such as wavelength^12–18^, polarization^19–25^, and phase^26–29^, have substantially shown great potential to replace the above redundant and complex systems in an easily-integrated and multifunctional way. Besides, multilayer configuration provides another wonderful platform to flexibly and efficiently construct metasurfaces. It allows more degrees of freedom to manipulate dimension of photons. Some significant advances have been reported based on multilayer metasurfaces^30–34^. Pioneering metadevices which surpass the traditional architectures in areas such as light splitting or polarization multiplexing have also been realized^35–39^. Notably, trichromatic hologram is achieved through the full-phase control at three discrete wavelengths by independently changing the geometry of the corresponding metaatoms^17,27,28,40–44^. Small crosstalk between neighboring detecting wavelengths, variable orthogonal polarization form over each wavelength, and high efficiency are always desired.

Herein, to break the eigen-polarization constraint we propose the dispersive Jones matrix method through constructing wavelength-decoupled coherent pixel based on all-silicon metasurface. Without spatial or time cost and crosstalk, this method enables the independent phase manipulations on any desired orthogonal polarization channels at predefined discrete wavelengths. It broadens the polarization applications from scientific research to industry in an ultra-compact and integratable manner which would require various cascaded elements in parallel otherwise.

## Results

### Design principle

As shown in Fig. 1b, the EPS can be accordingly adjusted through rotating the metaatoms at one single wavelength. Together with propagation phase control, the flexible metaatoms rotation enables the full phase manipulation in ref. ^19^ over one pair of arbitrarily selected orthogonal polarizations *P*_1_ and *P*_1_* at *λ*_1_. However, the simultaneous full control over another pair of arbitrary selected orthogonal polarizations *P*_2_ and *P*_2_* at *λ*_2_ further requires that the metaatom with rotation angle *θ*_2_ should satisfy two different phase shifts at *λ*_1_ and *λ*_2_ at the same time. Although this constraint can be alleviated by optimizing metaatom’s parameters other than the rotation angle (e.g., out-of-plane height or in-plane long/short axes), it would be challenging, if not impossible, to simultaneously and independently control phase over two (and more than two) pairs of orthogonal polarizations at different wavelengths, since the conversion path (and its related phase shift) for *P*_1_–*P*_1_* would differ from that for *P*_2_–*P*_2_* as Fig. 1b shows. For more pairs of orthogonal polarizations *P*_*n*_ and *P*_n_*, the eigen-polarization restrictions on the phase control becomes more critical. Until now, there is still no work reporting on the phase manipulations over more than two different pairs of orthogonal polarizations at multiple wavelengths.

In order to break the inherent eigen-polarization constraint for polarization control over different wavelengths, we propose a dispersive Jones matrix method through constructing wavelength-decoupled coherent pixel with four coherent all-silicon metaatoms in Fig. 1c. Generally, the zero-order diffraction efficiency would decrease with period larger than the operation wavelength. The nonuniform arrangement of metaatoms relieves this. More importantly, to guarantee the high performance of supercells operating at all three wavelengths and six polarization channels, the period the geometric parameters of the metaatoms are accordingly optimized to maximize the transmission and cover large phase controlling range through algorithms. The first-order diffraction at 3 μm is negligible. With this method, EPS of the pixel at different wavelengths can be adjusted accordingly to the orthogonal polarization control requirement.

To demonstrate this method, we start with the general form of Jones matrix for an elliptic cylinder metaatom given by^26^

1 $$J(\lambda ) = R\left( { - \theta } \right)\left( {\begin{array}{*{20}{c}} {e^{i\varphi _x(\lambda )}} & 0 \\ 0 & {e^{i\varphi _y(\lambda )}} \end{array}} \right)R\left( \theta \right)$$

where $$\scriptstyle{R\left( \theta \right) =\left[ {\begin{array}{*{20}{c}} {\cos \theta } & {\sin \theta } \\ { - \sin \theta } & {\cos \theta } \end{array}} \right]}$$ is a real unitary matrix corresponding to the in-plane geometrical rotation operation and determines the EPS of the metaatom. *θ* is the rotation angle and keeps constant with wavelength variation. The Jones matrix of our wavelength-decoupled coherent pixel can be written as:

2 $$\tilde J\left( \lambda \right) = \mathop {\sum }\limits_{k = 1}^4 R\left( { - \theta _k} \right)\left( {\begin{array}{*{20}{c}} {e^{i\varphi _x(\lambda )}} & 0 \\ 0 & {e^{i\varphi _y(\lambda )}} \end{array}} \right)R\left( {\theta _k} \right) = \left[ {\widehat {{{{\boldsymbol{e}}}}_1}\left( \lambda \right),\;\widehat {{{{\boldsymbol{e}}}}_2}\left( \lambda \right)} \right]^{ - 1}\left( {\begin{array}{*{20}{c}} {A(\lambda )} & 0 \\ 0 & {D(\lambda )} \end{array}} \right)\left[ {\widehat {{{{\boldsymbol{e}}}}_1}\left( \lambda \right),\;\widehat {{{{\boldsymbol{e}}}}_2}\left( \lambda \right)} \right]$$

where *k* stands for the index of different metaatoms in one supercell.

In this case, the original *J*(*λ*) in Eq. (1) has an EPS ${{{\hat{\boldsymbol e}}}} = \left[ {\cos \theta ,\sin \theta } \right]^T$, which is wavelength *λ* invariant. While the new *J* ~(*λ*) has a wavelength related EPS $\left[ {\widehat {{{{\boldsymbol{e}}}}_1}\left( \lambda \right),\;\widehat {{{{\boldsymbol{e}}}}_2}\left( \lambda \right)} \right]$, where the connection between the EPS and *λ* is broken. With this arrangement, the EPS of metaatoms over wavelength dimension are accordingly broken on the right side of Eq. (2). Relevant discussions on the constraint and decoupling disposal of fixed EPSs over wavelength dimension are shown in Supplementary Notes 1 and 2.

In Fig. 1d, a representative polychromatic full-polarization control metadevice is demonstrated to generate achromatically focusing spots over three pairs of arbitrarily chosen orthogonal polarizations on spatially separated channels at three wavelengths. It mimics the function of cascading filter, polarizer, and wave plate placed in parallel in conventional setups. Three different pairs of orthogonal polarization states at three wavelengths are linear polarization at 3.0 μm, elliptical polarization (ellipse angle 30°) at 3.6 μm and circular polarization at 4.5 μm. The distribution of phase retardation for this case is as follows:

3 $$\varphi _0\left( {j,k} \right) = - \frac{{2\pi }}{{\lambda _k}} \ast \left[ {\sqrt {\left( {x - a \ast \cos \vartheta } \right)^2 + \left( {y - a \ast \sin \vartheta } \right)^2 + f^2} - f} \right]$$

where *a* is the distance of focus spots to the origin, $\vartheta = \frac{{3 \ast \left( {j - 1} \right) + k - 1}}{3}\pi$ is the polar angle in focal plane, and *j* = 1, 2 represents the polarization state and its orthogonal polarization state, respectively. *k* = 1, 2, 3 is the operation wavelength. With this arrangement, the six-channel focal spots would evenly distribute along the circle with radius *a* at angle interval *π*/3.

### Optimization and implementation of the metadevice

It should be noted that there are 12 planar degrees of freedom in one wavelength-decoupled coherent pixel, i.e., the dimensions along long axes ($a_i$), short axes ($b_i$), and rotation angle ($\theta _i,\;i = 1,\;2,\;3,\;4$). The optimization space is 4th power of that in one traditional metaatom. Therefore, we develop an evolution algorithm combined with particle swarm optimization algorithm and genetic algorithm to find the supercells which matches best to the desired phases (more details can be found in Supplementary Note 3).

The objective function of optimization can be expressed as:

4 $$f_m = \mathop {\sum }\limits_{j,k} \left( {{{{\mathcal{R}}}}e\left\{ {\left( {\mathop {\sum }\limits_n J\left( {k,n} \right) \ast q\left( {j,k} \right)} \right)^ \ast } \right\}^T \ast e^{i{{{\mathrm{\varphi }}}}_0\left( {j,{{{\mathrm{k}}}}} \right)} \ast q(j,k)^ \ast } \right)$$

where *n* = 1, 2, 3, 4 represents the four metaatoms. *J* and *q* are the Jones matrix of a single metaatom and the desired polarization states.

**Fig. 2:**
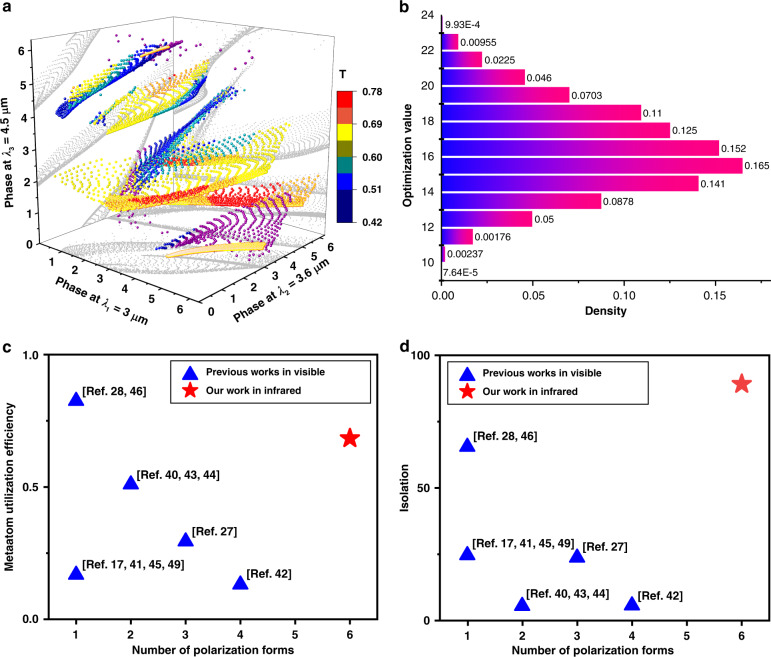
Optimization and comparison of the metadevice with reported works. **a** Three-dimensional phase dataset through sweeping the geometric dimensions at selected three wavelengths. The colors represent the average transmittance at three wavelengths. Gray points on the side and bottom surfaces represent the phases that simultaneously satisfy two wavelengths. **b** Merit function values after the evolution algorithm optimization process for the designed metadevice. The density represents the percentage of metaatoms that reach the corresponding optimization value. The maximum merit value 24 origins from the six-channel polarization states and four metaatoms in one supercell. **c** Efficiency comparison of the metadevice designed with our method with those reported in interleaved/segmented configurations at more than two operation wavelengths. There are no works reporting more than three wavelengths. **d** Comparison of isolation ratio between neighboring polarization channels for our metadevice and reported works in realizing functions in Fig. 1d

Three-dimensional phase coverage at designated wavelengths in the database is plotted in Fig. 2a. The color indicates the average transmittance. The phase optimization process is to find the best values in the database to approach the desired ones. To be more intuitive to evaluate the optimization, the density as function of optimization values is illustrated in Fig. 2b. The maximum value of the optimization function $f_m$ is 24. Higher optimization values indicate the better performance of the supercells. More than 80% phase values with optimization value larger than 14 are achieved here.

In order to have a straightforward comparison with previously reported metadevices operating at more than two wavelengths^17,27,28,35,40–49^, the function in Fig. 1d is reproduced with reported methods based on the built all-silicon metaatoms in this work. Figure 2c shows the efficiencies comparison. Efficiency is defined as the average optimal value divided by the theoretical maximum value. For interleaved/segmented method, the maximum efficiency is inversely proportional to the multiplexed channel. It can be seen that the efficiencies with our method are higher than all those reported, especially for those at three or even five wavelengths. Notably, we report the first realization of arbitrarily selected polarization-multiplexing over more than three wavelengths.

Isolation ratio is also used to demonstrate the merit of the dispersive Jones matrix method. It is defined as $Iso_{i,j} = \frac{{I_{i,i}}}{{I_{i,j}}},\;(i,j = 1,\;2, \ldots ,\;i \ne j)$, where *I* represent the intensity, *i* is the focal spot of design polarization state, *j* is the rest. It represents the ratio of the focal intensity on the desired polarization state to the other polarization states in the background, which directly indicates the decoupled properties between different polarization channels. Here, we averaged the isolation for all polarization channels in Fig. 2d. Details can be found in Supplementary Note 4 for the comparison of interleaved/segmented. Although there is still some space for the performance improvement with interleaved/segmented design methods, the inherent limitation of multiplexing the polarization channels over wavelength dimension makes it impossible to achieve high efficiency and polarization isolation ratio like ours.

### Experiments and characterization of the metadevice

**Fig. 3:**
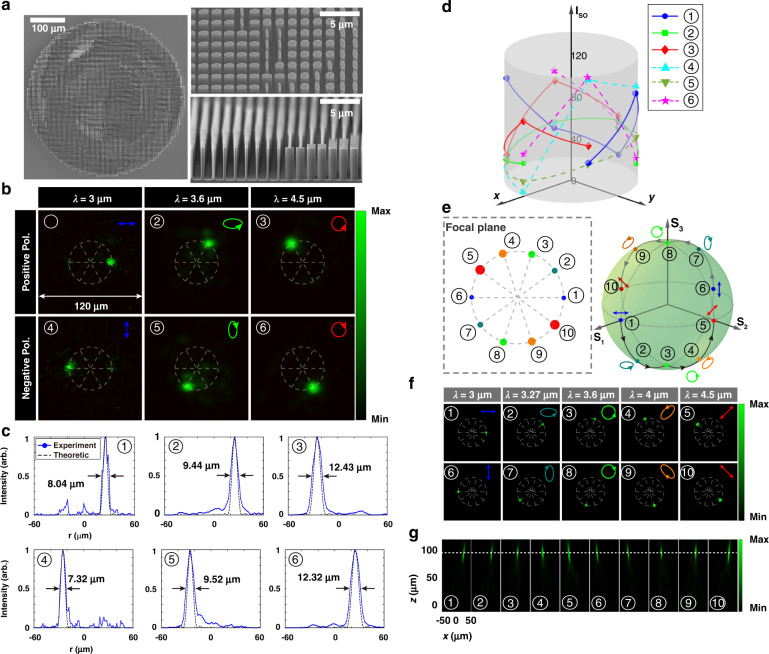
Experimental results of the metadevice. **a** SEM images from different angles of view. The metadevice is manufactured on a 500-μm-thick double-polished silicon wafer. **b** Captured images on the six polarization cannels numbered from 1 to 6. The experimental results are in consistent with the designated ones in Fig. 1d. The size of the images is 120 × 120 μm^2^. **c** Normalized intensity profiles of the six focal spots along the radially crossed lines. Measured FWHMs (solid blue) and theoretical Airy profiles (dashed black) are both depicted for comparison. **d** Isolation is utilized to characterize the decoupling property of the desired polarization from the other polarization channels. The colors of curves indicate the operating wavelengths, and the positions in the polar coordinate represent the locations of focal spots with specified polarization states. **e** Schematic of the focused spots on the focal plane for five operation wavelengths. The designated five independent pairs of orthogonal polarization states corresponding to five wavelengths are also depicted in the Poincaré sphere. **f** Focusing spots distributions on the focal plane for different polarizations at the selected five wavelengths. The positions are in consistent with those indicated in (**e**). The insets show the corresponding polarization states. **g** Intensity distributions on the cross-section planes

Experimental results for measured intensity on the image plane on each polarization channel are illustrated in Fig. 3b, which coincide well with the design target. To further evaluate the operating performance, full widths at half maximum (FWHMs) derived from intensity distributions are shown in Fig. 3c, showing that they fit well with the theoretical Airy profile. The measured focusing efficiencies are 36% (x-pol at 3 μm), 58.4% (left-handed elliptical polarization at 3.6 μm), 74.68% (left-handed circular polarization at 4.5 μm), 36.47% (y-pol at 3 μm), 53.34% (right-handed elliptical polarization at 3.6 μm), and 76.78% (left-handed circular polarization at 4.5 μm) respectively. It reveals the well performance of the designed metasurface. Though the metadevices are elaborately optimized to operate at single wavelength, in real cases the devices have bandwidths. Therefore, for a given wavelength range, the upper operation wavelength channel number is limited by the bandwidth. To illustrate this, we calculate the bandwidths for the metadevice with size 200 μm × 200 μm and focal length 400 μm in Supplementary Note 6. It’s also worth noting that more operation wavelength channels impose more restrictions on the dispersion of individual supercells. The optimization and searching for matched supercells would be more complicated.

The decoupling property represented by the contrast ratio is shown in Fig. 3d. For example, the red dot line represents the ratio of the average intensity at (1, 2π/3) (polar coordinate) to that of the other five positions (θ = π, 4π/3, 5π/3, 0, π/3). It can be seen that all the isolation ratios are greater than 10, with the highest one up to 117, manifesting the excellent decoupling performance of the six channels using our method. Compared with reported works for circular polarization control where the isolation ratio is less than 10^50–52^, our work not only achieves a circular polarization isolation ratio up to 96. which is one order of magnitude higher. We can also simultaneously engineer other arbitrarily selected orthogonal polarizations over other wavelengths.

Though the method is based on the wavelength-decoupled coherent pixel-each with only four conventional linear EPS metaatoms, it still works at multiple wavelengths and the form of polarization states are arbitrary. As an example, a ten-channel metadevice is realized as illustrated in Fig. 3e. The selected five pairs of orthogonal polarization states are shown in the Poincaré sphere. Simulation results on each polarization channel are respectively illustrated in Fig. 3f, which agrees well with the design. As predicted theoretically, ten focal spots on different polarization states are projected to the same focal plane, as shown in Fig. 3g, with the focal length almost unchanged at different wavelengths.

### Polychromatic vortex beams generation

**Fig. 4:**
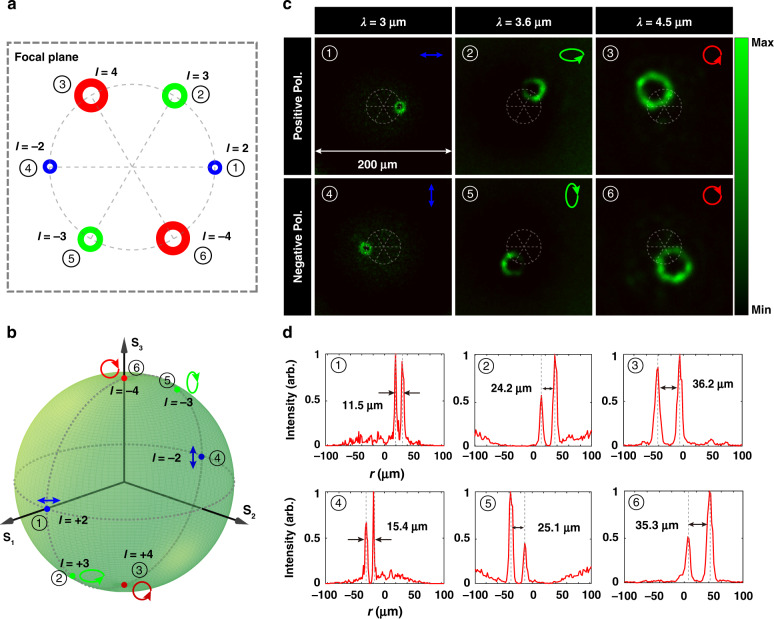
Characterizations of the metadevice for the generation of vortex beams. **a** Schematic of vortex beams on the Poincaré sphere. Three pairs of orthogonal polarization states endowed with a specified topological charge at three wavelengths are projected to specific positions numbered 1–6 on the focal plane. **b** The designated polarization states with different topological charge numbers are depicted on the Poincaré sphere. **c** Experimental results of the intensity distributions with six polarization channels. Distinct spots positions and intensity profiles coincide well with those in (**a**). The sizes of images are 200 × 200 μm^2^. **d** Normalized intensity profiles along radially-crossed lines. Different hollow sizes of the doughnut spots reveal that the carried topological charge number is different in each polarization channel

To illustrate the versatile engineering capability in implementing complicated and non-degenerate functionalities on each polarization channel, a polychromatic optical vortex beams generator is presented. In this case, each orthogonal polarization channel is endowed with a specified topological charge as shown in Fig. 4a. Vortex beams carrying distinct topological charges on each orthogonal polarization channel at different wavelengths are focused at positions 1–6: 1 represents the topological charge *l* = 2 at 3 μm on *x-polarization* (blue), 2 represents the topological charge *l* = 3 at 3.6 μm on *left-elliptical polarization* (green), 3 represents the topological charge *l* = 4 at 4.5 μm on *left-circular polarization* (red), 4 represents the topological charge *l* = −2 at 3 μm on *y-polarization* (blue), 5 represents topological charge *l* = −3 at 3.6 μm on *right-elliptical polarization* (green), and 6 represents the topological charge *l* = −4 at 4.5 μm on *right-circular polarization* (red). The polarization channels are also depicted on the Poincaré sphere, as shown in Fig. 4b.

The required phase modulations to fulfill the designated functions on each polarization state can be expressed as

5 $$\varphi \left( {j,k} \right) = - \frac{{2\pi }}{{\lambda _k}} \ast \left[ {\sqrt {\left( {x - a \ast \cos \theta } \right)^2 + \left( {y - a \ast \sin \theta } \right)^2 + f^2} - f} \right] + l \ast \Theta$$

where $l = \left( { - 2j + 3} \right) \ast (k + 1)$ is the topological charge number, and $\Theta = \arctan \frac{y}{x}$ is the azimuthal angle, with $(x,\;y)$ as the spatial coordinate for the metaatom on the metasurface plane. The evolution algorithm is also adopted to optimize the geometric dimensions and orientations of the metaatoms. Details on the optimization values and simulation results of the metadevice can be found in Supplementary Note 7.

Figure 4c illustrates the experimental results on the predefined polarization channels, which are in well coincidence with the designed functions in Fig. 4a. Different doughnut-beams on the specified polarization channel are observed on the designated positions. For each incident polarization, only one optical vortex on the conjugate polarization channel is attained on the focal plane. Figure 4d shows the normalized intensity profiles of the generated vortex beams on the cutting lines along radial directions. Optical vortices are projected to the predefined positions along the circle, and the hollow diameters vary with the topological charge number and operating wavelength.

### Polarization imaging with designed metasurface

To further explore the polarization imaging potentials of the method, firstly, we elaborately designed an object as the target image that consists of three subwavelength gratings to selectively reflect the specified incident polarization state at one wavelength while allow the other two polarizations to transmit at other wavelengths. Three key elements of the pattern are ‘stones’, ‘panda’, and ‘bamboo’, which respectively operate at wavelengths of 3, 3.6, and 4.5 μm. The full description of the scene is that a panda sitting on the stones is eating a bamboo. The design details on the pattern dimensions, fabrications, and transmissions of the gratings can be found in Supplementary Note 8. Accordingly, based on the dispersive Jones matrix method a metadevice with diameters of 1 mm is fabricated. Focusing functions are endowed to the designed polarization states at three desired wavelengths, i.e., *x-polarization* at 3 μm (blue), *y-polarization* at 3.6 μm (green), and *x-polarization* at 4.5 μm (red). It should be noted that the other three orthogonal polarizations are deliberately designed to diverge. The optimization process and the experimental characterization details of the metadevice can also be found in Supplementary Note 8.

**Fig. 5:**
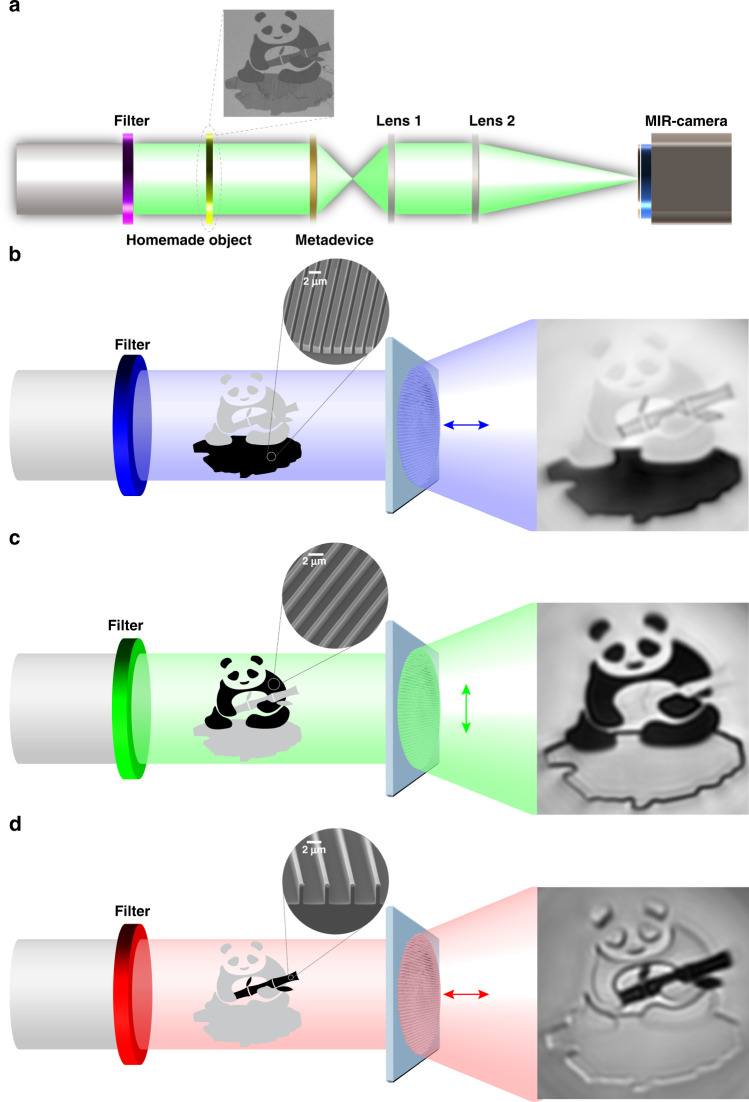
Imaging performance of the designed metadevice on charactering the panda pattern. **a** Setup of the imaging system, in which the object pattern consists of three subwavelength gratings. The inset shows the SEM image of the panda pattern. **b**–**d** Experimental results of the imaging. The measurements are successively conducted with the change of narrow bandpass filters. The insets show the SEM images of fabricated gratings. The operation wavelengths and polarization states are 3 μm *x-polarization*, 3.6 μm *y-polarization*, and 4.5 μm *x-polarization*. The light on other orthogonal polarization channels is deliberately designed to diverge

To demonstrate the imaging performance, we conduct the measurement with the setup shown in Fig. 5a. The experimental imaging results are illustrated with the order of 3 μm *x-p*, 3.6 μm *y-p*, 4.5 μm *x-p* in Fig. 5b–d, respectively. The color of the light indicates the operating wavelength and polarization state. With the metadevice, the underlying polarization information carried at different wavelengths can be independently distinguished. For example, only the bare stone shows up at the wavelength of 3 μm. The panda also emerges at the wavelength of 3.6 μm. Moreover, the panda sitting on the stones is carrying and eating a bamboo through the observation at the wavelength of 4.5 μm. Without this polychromatic polarization metasurface, series of conventional polarizers and lens are required to recognize the complex information. It is worth mentioning that this method also applies to more complex polarization states and wavelengths. This compact and versatile behavior of metadevices based on the dispersive Jones matrix provides a powerful platform to realize independent polarizations control. Besides, more patterns are fabricated and measured to further verify the feasibility of the metadevice, as shown in Supplementary Note 9. Particularly, to reflect the capability of generating holograms with our method, the three-wavelength 6-channel holograms of the mid-infrared are shown in Supplementary Note 10.

In the reported work^53^, authors introduce the engineered noise to the solution of Jones matrix and thus break the fundamental limit of polarization multiplexing of metasurfaces. 11 independent holographic images are demonstrated. Nonlocal metasurface designs provides a rational method to independent control of quasi-BICs at several discrete wavelengths^54,55^. This nonlocal paradigm greatly broadens the scope of metasurface design and enhances the capability to control both the spatial and spectral properties of light. As to our work, we propose the dispersive Jones matrix method through constructing wavelength-decoupled coherent pixel. The metaatoms in each supercell operates individually. The superposition of four Jones matrices enables the breaking of eigen-polarization and allows the realization of multiple functions in six different polarization channels at three wavelengths.

## Discussion

In this work, we propose a dispersive Jones matrix method through constructing wavelength-decoupled coherent pixel based on all-silicon metasurface. Eigen-polarizations at different wavelengths can be engineered. This method enables the independent phase manipulations on any desired orthogonal polarization channels at predefined discrete wavelengths. We realized the full-polarization control at multiple discrete wavelengths. Remarkably, the diverse orthogonal polarization states are experimentally demonstrated over more than three wavelengths. Conventional metasurfaces constructed with interleaved/segmented approaches can realize either wavelength or polarization multiplexing. Neighboring metaatoms have interactions which would lead to the crosstalk between operating wavelength/polarization channels and/or the decreases in efficiency. The polarization forms at multiplexing wavelengths are the same since the eigen-polarizations are the same at different wavelengths. In this work, the adoption of supercells allows the engineering of eigen-polarizations at different wavelengths. Simultaneously arbitrary polarization multiplexing at multiple wavelengths are realized. Besides, the optimized supercells help to improve the channel isolations. Low-temperature deep silicon etching technology at −150 °C guarantees the high aspect ratio and uniform fabrication, allowing versatile and precise manipulation of metaatoms in realizing complex functions. Immune from complicated cascaded configurations, this method enables multiple functions in one single metasurface and opens possibilities to engineer the unconventional birefringent phase profiles, i.e., simultaneously imparting any desired and independent orthogonal polarization states to any predefined discrete wavelengths. The versatility of the dispersive Jones matrix method is beneficial for specific applications in airborne/space-borne onboard payloads in a low-illumination environment, for instance, the polarization imaging at multiple wavelengths in an ultra-integrated and multifunctional fashion.

## Materials and methods

### Sample fabrication

To experimentally demonstrate the designed metadevice, an all-silicon metasurface is fabricated with electron beam lithography at accelerating voltage 100 kV (Jeol 6300FS). A 50-nm-thick aluminum film is adopted as the hard mask on one side of 4-inch double-polished silicon wafer. Remarkably, benefiting from the liquid nitrogen and helium back-cooling control technology, metadevices with almost perfect profiles are obtained with deep silicon etching machine (Estrelas 100) operating at −150 °C. It guarantees the highest aspect ratio up to 30 and ensures that metaatoms of different aspect ratios are uniformly fabricated with perfect sidewalls. The whole size of metadevice is 400 μm in diameter, and the height of the metaatoms is 6 μm. The minimum feature linewidth of structures is 250 nm with the neighboring distance larger than 300 nm, as shown in the scanning electron microscopes (SEM) images in Fig. 3a. The tilted and cross-section views of the zoom-in metaatoms manifest the excellent homogenous fabrications, especially for metaatoms with different aspect ratios.

### Measurement setup

The measurement system to characterize the metadevice is shown in Supplementary Materials Fig. S6. The blackbody which is a broadband thermal radiator, is adopted as the light source. The linear polarizer and liquid crystal retarder (LCC1113-MIR) are used to modulate the polarizations of incident light. In the light path, the sample is vertically fixed on the hollow acrylic sample rack. It can be finely adjusted for alignment and focus through tuning the six-axis translation and rotation stage. The microscopic module is composed of a 4-mm aspheric lens and a 25-mm lens to magnify the focal spots. The transmitted light after interacting with the metadevice is then captured by the mid-wave infrared camera which is cooled at around 80 K with Stirling cryocooler. It’s worth noting that narrow bandpass filters are adopted to keep the coherence of blackbody radiation both in time and space in the measurement.

## Supplementary information

SUPPLEMENTAL MATERIAL

Polychromatic polarization metalens

Polychromatic vortex beams generation

Polychromatic polarization imaging

## References

[1] Nasrabadi NM (2014). Hyperspectral Target Detection: an overview of current and future challenges. IEEE Signal Process. Mag..

[2] Vanderbilt VC, Grant L, Daughtry CST (1985). Polarization of light scattered by vegetation. Proc. IEEE.

[3] Deschamps PY (1994). The POLDER mission: instrument characteristics and scientific objectives. IEEE Trans. Geosci. Remote Sens..

[4] Case TW (1917). Notes on the change of resistance of certain substances in light. Phys. Rev..

[5] Lawson WD (1959). Preparation and properties of HgTe and mixed crystals of HgTe-CdTe. J. Phys. Chem. Solids.

[6] Bayer, B. E. *Color Imaging Array* (1976).

[7] Stocker, A. D. et al. Analysis of infrared multispectral target/background field measurements. *Proceedings of SPIE 2235, Signal and Data Processing of Small Targets 1994* 148–161 (SPIE, 1994).

[8] Stocker, A. D. et al. Analysis of infrared hyperspectral measurements by the joint multispectral program. *Proceedings of SPIE 2469, Targets and Backgrounds: Characterization and Representation* 587–602 (SPIE, 1995).

[9] Sposato SH (2002). Two long-wave infrared spectral polarimeters for use in understanding polarization phenomenology. Opt. Eng..

[10] Garlick, G. F. J., Steigmann, G. A. & Lamb, W. E. *Differential Optical Polarization Detectors* (1976).

[11] Matchko RM, Gerhart GR (2008). High-speed imaging chopper polarimetry. Opt. Eng..

[12] Sun SL (2012). Gradient-index meta-surfaces as a bridge linking propagating waves and surface waves. Nat. Mater..

[13] Ni XJ, Kildishev AV, Shalaev VM (2013). Metasurface holograms for visible light. Nat. Commun..

[14] Yu NF, Capasso F (2014). Flat optics with designer metasurfaces. Nat. Mater..

[15] Aieta F (2015). Multiwavelength achromatic metasurfaces by dispersive phase compensation. Science.

[16] Khorasaninejad M (2016). Metalenses at visible wavelengths: diffraction-limited focusing and subwavelength resolution imaging. Science.

[17] Wang B (2016). Visible-frequency dielectric metasurfaces for multiwavelength achromatic and highly dispersive holograms. Nano Lett..

[18] Chen WT (2018). A broadband achromatic metalens for focusing and imaging in the visible. Nat. Nanotechnol..

[19] Balthasar Mueller JP (2017). Metasurface polarization optics: independent phase control of arbitrary orthogonal states of polarization. Phys. Rev. Lett..

[20] Rubin NA (2019). Matrix Fourier optics enables a compact full-Stokes polarization camera. Science.

[21] Zhang XQ (2019). Direct polarization measurement using a multiplexed Pancharatnam–Berry metahologram. Optica.

[22] Fan QB (2020). Independent amplitude control of arbitrary orthogonal states of polarization via dielectric metasurfaces. Phys. Rev. Lett..

[23] Shi ZJ (2020). Continuous angle-tunable birefringence with freeform metasurfaces for arbitrary polarization conversion. Sci. Adv..

[24] Bao YJ (2021). Toward the capacity limit of 2D planar Jones matrix with a single-layer metasurface. Sci. Adv..

[25] Dorrah AH (2021). Metasurface optics for on-demand polarization transformations along the optical path. Nat. Photonics.

[26] Arbabi A (2015). Dielectric metasurfaces for complete control of phase and polarization with subwavelength spatial resolution and high transmission. Nat. Nanotechnol..

[27] Hu YQ (2020). Trichromatic and tripolarization-channel holography with noninterleaved dielectric metasurface. Nano Lett..

[28] Deng ZL (2020). Full‐color complex‐amplitude vectorial holograms based on multi‐freedom metasurfaces. Adv. Funct. Mater..

[29] Jang M (2018). Wavefront shaping with disorder-engineered metasurfaces. Nat. Photonics.

[30] Chang T (2022). Universal metasurfaces for complete linear control of coherent light transmission. Adv. Mater..

[31] Zhou Y (2018). Multilayer noninteracting dielectric metasurfaces for multiwavelength metaoptics. Nano Lett..

[32] Avayu O (2017). Composite functional metasurfaces for multispectral achromatic optics. Nat. Commun..

[33] Zhou Y (2019). Multifunctional metaoptics based on bilayer metasurfaces. Light Sci. Appl..

[34] Bao YJ (2022). Observation of full-parameter Jones matrix in bilayer metasurface. Nat. Commun..

[35] Chen C (2020). Parallel polarization illumination with a multifocal axicon metalens for improved polarization imaging. Nano Lett..

[36] McClung A (2020). Snapshot spectral imaging with parallel metasystems. Sci. Adv..

[37] Faraji-Dana M (2019). Hyperspectral imager with folded metasurface optics. ACS Photonics.

[38] Faraji-Dana M (2018). Compact folded metasurface spectrometer. Nat. Commun..

[39] Yu FL (2022). Photonic slide rule with metasurfaces. Light Sci. Appl..

[40] Jin L (2018). Noninterleaved metasurface for (2^6^-1) spin- and wavelength-encoded holograms. Nano Lett..

[41] Bao YJ (2019). Full-colour nanoprint-hologram synchronous metasurface with arbitrary hue-saturation-brightness control. Light Sci. Appl..

[42] Guo XY (2022). Full‐color holographic display and encryption with full‐polarization degree of freedom. Adv. Mater..

[43] Feng H (2019). Spin-switched three-dimensional full-color scenes based on a dielectric meta-hologram. ACS Photonics.

[44] Bao, Y. J. et al. Dielectric metasurface for independent complex-amplitude control of arbitrary two orthogonal states of polarization. Preprint at https://arxiv.org/abs/2105.13640 (2021).

[45] Hu YQ (2019). 3D-Integrated metasurfaces for full-colour holography. Light Sci. Appl..

[46] Shi ZJ (2018). Single-layer metasurface with controllable multiwavelength functions. Nano Lett..

[47] Yang ZY (2018). Generalized Hartmann-Shack array of dielectric metalens sub-arrays for polarimetric beam profiling. Nat. Commun..

[48] Arbabi E (2016). Multiwavelength polarization-insensitive lenses based on dielectric metasurfaces with meta-molecules. Optica.

[49] Huo PC (2020). Photorealistic full-color nanopainting enabled by a low-loss metasurface. Optica.

[50] Hubbs, J. E. et al. Measurement of the radiometric and polarization characteristics of a microgrid polarizer infrared focal plane array. *Proceedings of SPIE 6295, Infrared Detectors and Focal Plane Arrays VIII* 62950C (SPIE, 2006).

[51] Zhu AY (2018). Giant intrinsic chiro-optical activity in planar dielectric nanostructures. Light Sci. Appl..

[52] Li W (2015). Circularly polarized light detection with hot electrons in chiral plasmonic metamaterials. Nat. Commun..

[53] Xiong B (2023). Breaking the limitation of polarization multiplexing in optical metasurfaces with engineered noise. Science.

[54] Malek SC (2022). Multifunctional resonant wavefront-shaping meta-optics based on multilayer and multi-perturbation nonlocal metasurfaces. Light Sci. Appl..

[55] Overvig AC, Malek SC, Yu NF (2020). Multifunctional nonlocal metasurfaces. Phys. Rev. Lett..

