# Supplementary material for: Polychromatic full-polarization control in mid-infrared light
Source: Light Sci Appl. 2023 May 4;12:105. doi: 10.1038/s41377-023-01140-3 (PMC10160079; doi:10.1038/s41377-023-01140-3)
Supplement: Supplementary file 1 — SUPPLEMENTAL MATERIAL [file 41377_2023_1140_MOESM1_ESM.docx]

Supplementary Information for:

**Polychromatic full-polarization control in mid-infrared light**

Jin Chen^1,2,3,4,#^, Feilong Yu^1,2,3,4,#^, Xingsi Liu^5^, Yanjun Bao^6^, Rongsheng Chen^1,4^, Zengyue Zhao^1,4^, Jiuxu Wang^1,4^, Xiuxia Wang^7^, Wen Liu^7^, Yuzhi Shi^8^, Cheng-Wei Qiu^5,9^*, Xiaoshuang Chen^1,2,3,4^, Wei Lu^1,2,3,4^, and Guanhai Li^1,2,3,4,*^

^1^State Key Laboratory of Infrared Physics, Shanghai Institute of Technical Physics, Chinese Academy of Sciences, 500 Yu Tian Road, Shanghai, 200083, China

^2^Hangzhou Institute for Advanced Study, University of Chinese Academy of Sciences, No.1 SubLane Xiangshan, Hangzhou, 310024, China

^3^Shanghai Research Center for Quantum Sciences, 99 Xiupu Road, Shanghai, 201315, China

^4^University of Chinese Academy of Science, No.19 Yuquan Road, Beijing 100049, China

^5^Department of Electrical and Computer Engineering, National University of Singapore, 4 Engineering Drive 3, Singapore 117583, Singapore

^6^Institute of Nanophotonics, Jinan University, Guangzhou 511443, China

^7^Center for Micro-and Nanoscale Research and Fabrication, University of Science and Technology of China, Hefei, 230026, China

^8^Institute of Precision Optical Engineering, School of Physics Science and Engineering, Tongji University, Shanghai 200092, China

^9^National University of Singapore Suzhou Research Institute, No. 377 Linquan Street Suzhou, Jiangsu 215123, China

*Corresponding Email: [*ghli0120@mail.sitp.ac.cn*](mailto:ghli0120@mail.sitp.ac.cn)*;* [*chengwei.qiu@nus.edu.sg*](mailto:chengwei.qiu@nus.edu.sg)

Supplementary Note 1 Eigen-polarization state constraints on phase modulation

1. Direct solution to the polarization control at single wavelength

For an incident light with an arbitrary polarization state ${\hat{\boldsymbol{p}}}_{in}=\left[ \begin{aligned} \cos\alpha\\ \sin\alpha e^{i\beta} \end{aligned} \right]$ passing through a linear birefringent metaatom with orientation angle *θ*, the output can be written as

$$\begin{aligned} \begin{aligned} {\hat{\boldsymbol{p}}}_{out}&=R\left( -\theta\right)\left[ \begin{matrix} 1 & 0 \\ 0 & e^{i\delta} \end{matrix} \right]R\left( \theta\right)\left[ \begin{aligned} \cos\alpha\\ \sin\alpha e^{i\beta} \end{aligned} \right] \\ &=\left[ \begin{aligned} \cos\alpha\left( \cos^{2} \theta+\sin^{2} \theta e^{i\delta} \right)-\sin\theta\cos\theta\sin\alpha\left( e^{i\delta}-1 \right)e^{i\beta} \\ \sin\alpha\left( \sin^{2} \theta+\cos^{2} \theta e^{i\delta} \right)e^{i\beta}-\sin\theta\cos\theta\cos\alpha\left( e^{i\delta}-1 \right) \end{aligned} \right] \end{aligned}\#\left( \boldsymbol{s}\boldsymbol{1} \right) \end{aligned}$$

For conjugate polarizations control, it needs

$$\begin{aligned} {\hat{\boldsymbol{p}}}_{out}=e^{i\varphi}\cdot\left[ \begin{aligned} \cos\alpha\\ \sin\alpha e^{-i\beta} \end{aligned} \right]\#\left( \boldsymbol{s}\boldsymbol{2} \right) \end{aligned}$$

With **Eqs. (s1)** and **(s2)**, *δ* and *φ* can be solved with the following expressions:

$$\begin{aligned} \begin{aligned} e^{i\delta}&=\frac{\sin2\alpha\left( \cos^{2} \theta e^{-i\beta}-\sin^{2} \theta e^{i\beta} \right)-\sin2\theta\cos2\alpha}{\sin2\alpha\left( \cos^{2} \theta e^{i\beta}-\sin^{2} \theta e^{-i\beta} \right)-\sin2\theta\cos2\alpha} \\ e^{i\varphi}&=e^{i\beta}\frac{\sin2\theta\sin^{2} \alpha e^{i2\beta}+\sin2\alpha\cos2\theta e^{i\beta}-\sin2\theta\cos^{2} \alpha}{\sin2\alpha\cos^{2} \theta e^{i2\beta}-\sin2\theta\cos2\alpha e^{i\beta}-\sin2\alpha\sin^{2} \theta} \end{aligned}\#\left( \boldsymbol{s}\boldsymbol{3} \right) \end{aligned}$$

where *δ* is the phase difference between the two linear eigen-polarizations basis and *φ* is the overall phase. With above equations, we can fully determine the polarization function of the designed metadevice. It is worth mentioning that the symmetry of conventional linear birefringent metaatoms imposes the mandatory choice of linear eigen-polarization basis.

In the following we will discuss the angular orientation restrictions on the polarization control from the point of space state view on the Poincaré sphere. It should be noted that for mirror symmetric structures, the basic parameters for modulations are the characteristic phase retardance *δ* and orientation angle *θ*. The phase retardance *δ* represents the central angle of precession routing on the Poincaré sphere and orientation angle *θ* represents the procession axis. Both *δ* and *θ* determine the precession routings and corresponding geometric phases. The most essential polarization engineering is the choice of desired orthogonal polarization channels and the control of geometric phases.

2. Orientation restriction on the polarization controlling channels

For a linear birefringent metaatom with a fixed orientation angle *θ*, once the incident polarization state is specified, the dimensions of the planar geometry can be adjusted to convert it to the conjugate polarization along a route which is determined by the orientation and conjugate polarizations as shown in **Fig. s1a**. In this condition, *δ* is also fixed. Therefore, another pair of arbitrary conjugate polarizations conversion is impossible due to the exhausted geometric degrees of freedom. In other words, despite different pair of conjugate polarizations can be fulfilled at one time through tailoring the phase retardance *δ*, no geometric parameters are available to be varied to simultaneously realize the arbitrary overall phases *φ* anymore as shown in **Fig. s1b**. Therefore, the overall phase shift *φ* and the phase retardance *δ* is cross bonding at a fixed angular orientation. At one single wavelength, the polarization channels for the conventional linear birefringent metaatoms are limited to only one pair.


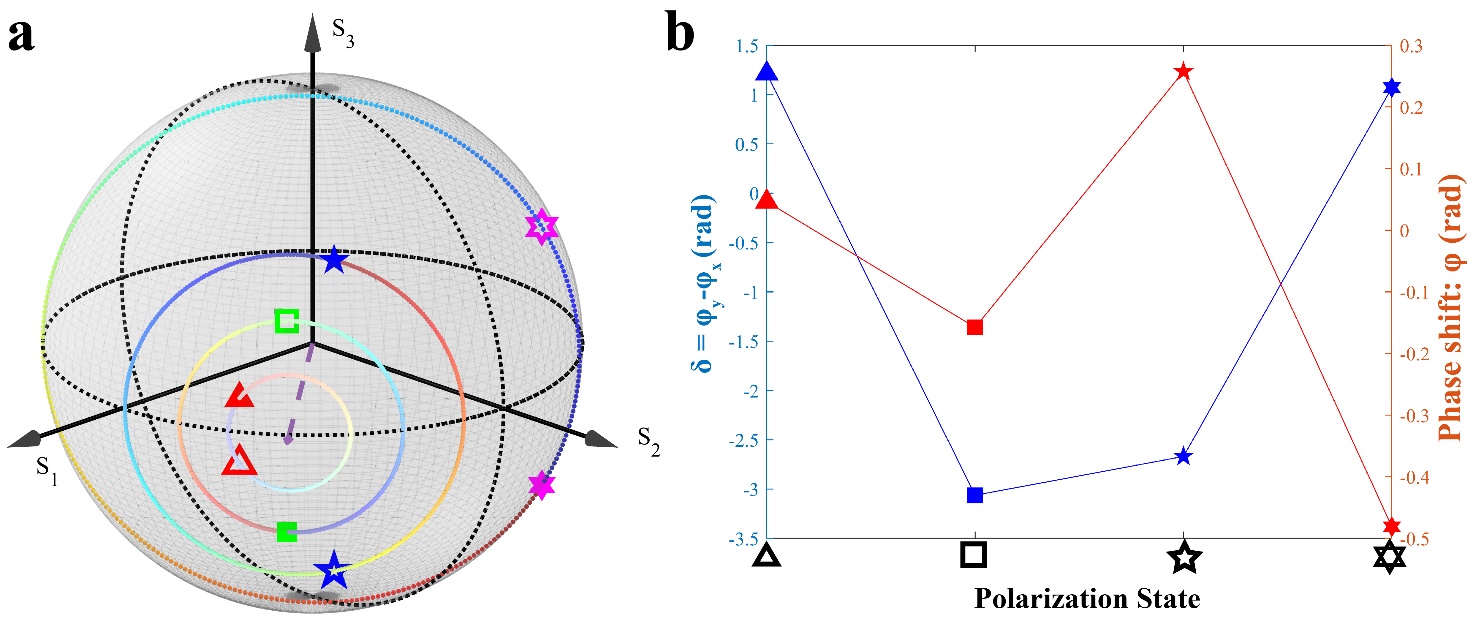


**Fig. s1** **a** Schematic of different conjugate polarization conversions for a fixed orientation angle at a single wavelength. **b** the characteristic phase difference and corresponding phase shift at different polarizations channels.

To overcome the orientation constraint on the operating polarization channels, the system symmetry is innovatively broken through utilizing different incident angles with a freeform metasurface. However, the phase engineering enabled functionalities on each polarization channel is unresolved.

3. Orientation restriction on the polarization transforming routings


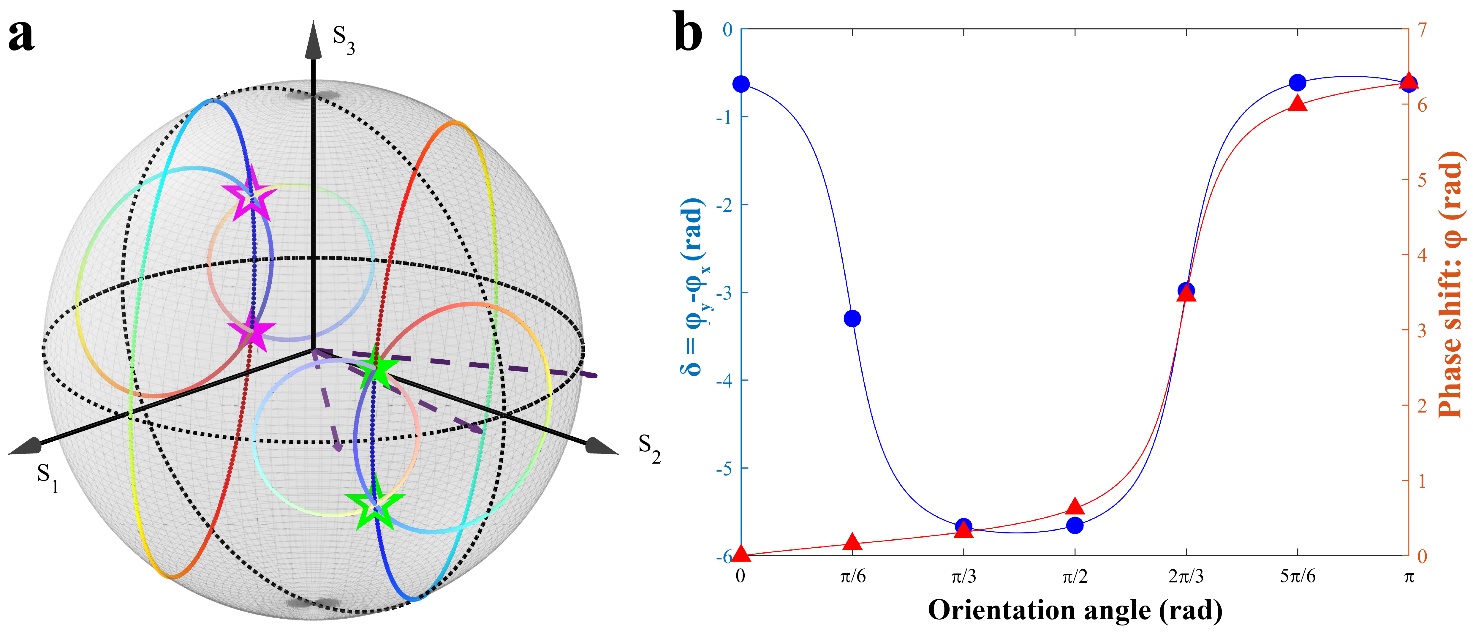


**Fig. s2 a** schematic of the different routings for conjugate polarizations conversions with a given incident polarization. **b** the phase retardance and overall phase shift as the angular orientation changes.

As shown in **Fig. s2a**, the precession axes are always pointing from the sphere center to the equator on the Poincaré sphere for linear eigen-polarization structures. Once orthogonal polarizations channels and the orientation are specified as indicated in **Fig. s2a,** the conjugate polarization conversion routing is solely determined along the circles which are defined by the conjugate polarizations states and the precession axes. The precession routing planes are normal to the precession axes. The corresponding geometric phases, one for the conjugate polarization conversion and the other one for its orthogonal polarization, are also determined. To achieve variable phase retardance *δ* and overall phases *φ*, the orientation angle *θ* can be rotated as shown in **Fig. s2a**. Different circles represent different precession routings. As marked with colors from blue to red, the phase retardance along the routings is accordingly changed from 0 to 2π. It can be seen that both the phase retardance *δ* and overall phases *φ* cover 2*π* range in **Fig. s2b**. However, for another pair of orthogonal polarizations channels, the determined precession routing and the fixed orientation limit each other to realize the fully independent control.

Supplementary Note 2 The dispersive Jones matrix method for breaking the angular orientation restriction

With above discussions, at single wavelength, the birefringent metaatom orientation restricts the simultaneous realization of multiple pairs independent orthogonal polarization channels and the arbitrarily selected precession routings. In other words, for symmetric birefringent structures which are based on linear eigen-polarization basis, the orthogonal polarizations channels and the geometric phases are bound to the wavelength dimension for a given angular orientation. Previous works mainly focus on the spatial modulations to decouple the polarization channels or geometric phases within the regulation of structure symmetry. In this work, through breaking the metaatoms symmetry, the method to decouple the orientation in the wavelength dimension provides a completely new avenue to engineer the orthogonal polarization channels and therefore enables independent geometric phase manipulations over different polarization channels simultaneously, which are of great significance in multiple wavelengths applications like hazardous gas detection or multicolor holography. It is worth mentioning that the intuitive way to solve this problem with the intrinsic dispersion of metaatoms is not possible considering the restricted geometric phases for different pairs of independent orthogonal polarization states at a fixed orientation angle for a given metaatom position, i.e., the independent phase coverage over different wavelengths at a given metaatom position is limited.

We will start with the arbitrary selection of eigen-polarization basis to introduce the design.

1. The eigen-polarization basis of conventional metaatoms

For a conventional linear birefringent metaatom like rectangular or elliptical nanostructures, the Jones matrix can be written as

$$\begin{aligned} J=\left[ \begin{matrix} e^{i\varphi_{x}} & 0 \\ 0 & e^{i\varphi_{y}} \end{matrix} \right]\#\left（ s4 \right） \end{aligned}$$

With the introduction of geometric phases, it can be modified as

$$\begin{aligned} \begin{aligned} J&=R\left( -\theta\right)\left[ \begin{matrix} e^{i\varphi_{x}} & 0 \\ 0 & e^{i\varphi_{y}} \end{matrix} \right]R\left( \theta\right) \\ &=\left[ \begin{matrix} \cos\theta& -\sin\theta\\ \sin\theta& \cos\theta\end{matrix} \right]\left[ \begin{matrix} e^{i\varphi_{x}} & 0 \\ 0 & e^{i\varphi_{y}} \end{matrix} \right]\left[ \begin{matrix} \cos\theta& \sin\theta\\ -\sin\theta& \cos\theta\end{matrix} \right] \end{aligned}\#\left( s5 \right) \end{aligned}$$

where $R\left( \theta\right)$ represents the eigen-polarization basis. For example, for a metaatom orientated at an angle *θ*, its eigen-polarization basis is <*θ*, *θ* + *π*/2>.

2. The arbitrary eigen-polarization basis realization

To decouple the angular orientation from the wavelength dimension and release its restrictions on the polarization channels and geometric phases, we propose the dispersive Jones matrix method through breaking the symmetry of the conventional metaatoms with composite supercells.

Then the overall Jones matrix can be written as the superpositions of four birefringent metaatoms

$$\begin{aligned} \begin{aligned} J&=\sum_{k=1}^{4} J_{k}=\left[ \begin{matrix} \sum_{k=1}^{4} A_{k} & \sum_{k=1}^{4} B_{k} \\ \sum_{k=1}^{4} C_{k} & \sum_{k=1}^{4} D_{k} \end{matrix} \right]=\left[ \begin{matrix} A & B \\ B & D \end{matrix} \right] \end{aligned}\#\left( s6 \right) \end{aligned}$$

where $A_{k}=\cos^{2} \theta_{k}e^{i\varphi_{x_{k}}}+\sin^{2} \theta_{k}e^{i\varphi_{y_{k}}}$, $B_{k}=C_{k}=\sin\theta_{k}\cos\theta_{k}\left( e^{i\varphi_{x_{k}}}-e^{i\varphi_{y_{k}}} \right)$ and $D_{k}=\sin^{2} \theta_{k}e^{i\varphi_{x_{k}}}+\cos^{2} \theta_{k}e^{i\varphi_{y_{k}}}$.

Two eigen values of **Eq. (s6)** are

$$\begin{aligned} \begin{aligned} k_{1,2}&=\frac{A+D\pm\sqrt{\left( A-D \right)^{2}+4B^{2}}}{2} \\ J&=\boldsymbol{\Lambda}\left[ \begin{matrix} k_{1} & 0 \\ 0 & k_{2} \end{matrix} \right]\boldsymbol{\Lambda}^{-1} \\ \boldsymbol{\Lambda&}=\left[ \begin{matrix} 1 & \frac{k_{2}-D}{B} \\ \frac{k_{1}-A}{B} & 1 \end{matrix} \right]=\left[ \begin{matrix} 1 & -\frac{X}{2B} \\ \frac{X}{2B} & 1 \end{matrix} \right] \end{aligned}\#\left( s7 \right) \end{aligned}$$

where $X=-\left( A-D \right)+\sqrt{\left( A-D \right)^{2}+4B^{2}}$.

The eigen vectors are the polarizations basis of the composite metaatoms.

$$\begin{aligned} \begin{aligned} \hat{\boldsymbol{e}_{\boldsymbol{1}}}=\left[ \begin{aligned} \cos R \\ \sin Re^{i\gamma} \end{aligned} \right], \hat{\boldsymbol{e}_{\boldsymbol{2}}}=\left[ \begin{aligned} -sin R \\ \cos Re^{-i\gamma} \end{aligned} \right] \\ \cos R=\frac{1}{\sqrt{1+F^{2}}}, \sin R=\frac{F}{\sqrt{1+F^{2}}} \end{aligned}\#\left( s8 \right) \end{aligned}$$

where $Fe^{i\gamma}=\frac{X}{2B}.$

It can be seen that the polarization basis of the supercell has been converted to the most general case-elliptical basis. Only at the condition *γ = 2n*π, *n* is an integer, the eigen-polarization basis degenerates to orthogonal linear ones.

With this disposal, the inherent restriction of orientation on the choosing of eigen-polarization basis is totally broken. The arbitrary selection of eigen-polarization basis allows us to realize multiple pairs of independent orthogonal polarizations channels and geometric phases manipulations in different wavelengths since the arbitrary precession routings and equivalent orientations can be fulfilled. It can also be concluded that the eigen-polarization states in **Eq. (s8)** are dependent on orientation angles $\theta_{k}$ and the phases ($\varphi_{x_{k}}$, $\varphi_{y_{k}})$ of four metaatoms. We can optimize 12 degrees of freedom with algorithms to freely determine the eigen-polarization basis and accordingly to engineer the metadevices functions.

3. The decoupling on the wavelength dimension

It should be noted that in this work we stick to the degenerated linear eigen-polarizations basis to demonstrate the method on wavelength decoupling and compare it with existing works. With this consideration, *γ* should equals 2*n*π according to **Eq. (s8)**. In order to realize full geometric phase control over arbitrarily selected polarization channels, the values of *R* should also be satisfied simultaneously.

According to **Eq. (s8)**, when *γ* = 0*, R* varies from 0 to π and can be modulated with the optimized combination of 12 degrees of freedom. Considering the complexity on solving $R=f\left( \theta_{k}, \varphi_{x_{k}}, \varphi_{y_{k}} \right), k=1, 2, 3, 4.$ We show some particular solutions in **Table s1** below.

**Table s1** Particular solutions of *R* as function of $\varphi_{x}$ and $\varphi_{y}$

| Eigenstate (rad)  Orientation  angle *θ* (degree) | Unit 1 | | Unit 2 | | Unit 3 | | Unit 4 | |
| --- | --- | --- | --- | --- | --- | --- | --- | --- |
|  | $\varphi_{x}$ | $\varphi_{y}$ | $\varphi_{x}$ | $\varphi_{y}$ | $\varphi_{x}$ | $\varphi_{y}$ | $\varphi_{x}$ | $\varphi_{y}$ |
| 0 | 0.90 | 0 | 0 | 6.28 | 3.99 | 1.69 | 6.28 | 0 |
| 5 | 2.82 | 4.39 | 1.31 | 1.89 | 0 | 4.77 | 2.87 | 2.16 |
| 10 | 3.81 | 3.93 | 6.28 | 6.28 | 4.78 | 4.97 | 2.77 | 0 |
| 15 | 4.35 | 2.44 | 6.28 | 2.32 | 1.77 | 5.19 | 1.22 | 3.39 |
| 20 | 5.69 | 4.03 | 2.28 | 4.49 | 0 | 1.95 | 4.99 | 5.54 |
| 25 | 3.98 | 5.92 | 0 | 6.28 | 0 | 6.28 | 0 | 3.53 |
| 30 | 0.22 | 6.11 | 0.23 | 6.10 | 3.48 | 2.24 | 4.71 | 1.00 |
| 35 | 3.54 | 0.87 | 5.30 | 6.28 | 4.51 | 0.44 | 5.20 | 2.29 |
| 40 | 2.98 | 0.99 | 3.02 | 4.70 | 3.02 | 3.36 | 1.36 | 0.02 |
| 45 | 0 | 0 | 0 | 0 | 0 | 0 | 6.28 | 3.14 |
| 50 | 3.53 | 0.16 | 4.30 | 3.99 | 1.02 | 5.30 | 0.40 | 0.68 |
| 55 | 5.35 | 0 | 6.28 | 2.48 | 3.94 | 0 | 2.78 | 6.15 |
| 60 | 6.28 | 6.05 | 2.71 | 0.82 | 6.28 | 6.17 | 0.08 | 6.17 |
| 65 | 4.78 | 1.84 | 2.48 | 4.25 | 4.96 | 4.89 | 2.83 | 4.80 |
| 70 | 3.51 | 0.17 | 0.67 | 3.04 | 2.80 | 1.96 | 2.27 | 2.02 |
| 75 | 0 | 2.03 | 0 | 0 | 3.62 | 0.02 | 0 | 2.90 |
| 80 | 3.64 | 2.33 | 1.01 | 4.92 | 2.02 | 3.63 | 0 | 4.78 |
| 85 | 1.17 | 6.28 | 5.47 | 6.28 | 3.76 | 3.35 | 6.28 | 3.76 |
| 90 | 1.57 | 4.71 | 6.28 | 0 | 6.28 | 6.28 | 6.28 | 6.28 |

For demonstration, we show three different equivalent orientation angle plots as a function of wavelength in **Fig. s3** to illustrate the flexibility of our method for breaking the orientation restrictions when extending over wavelength dimension.


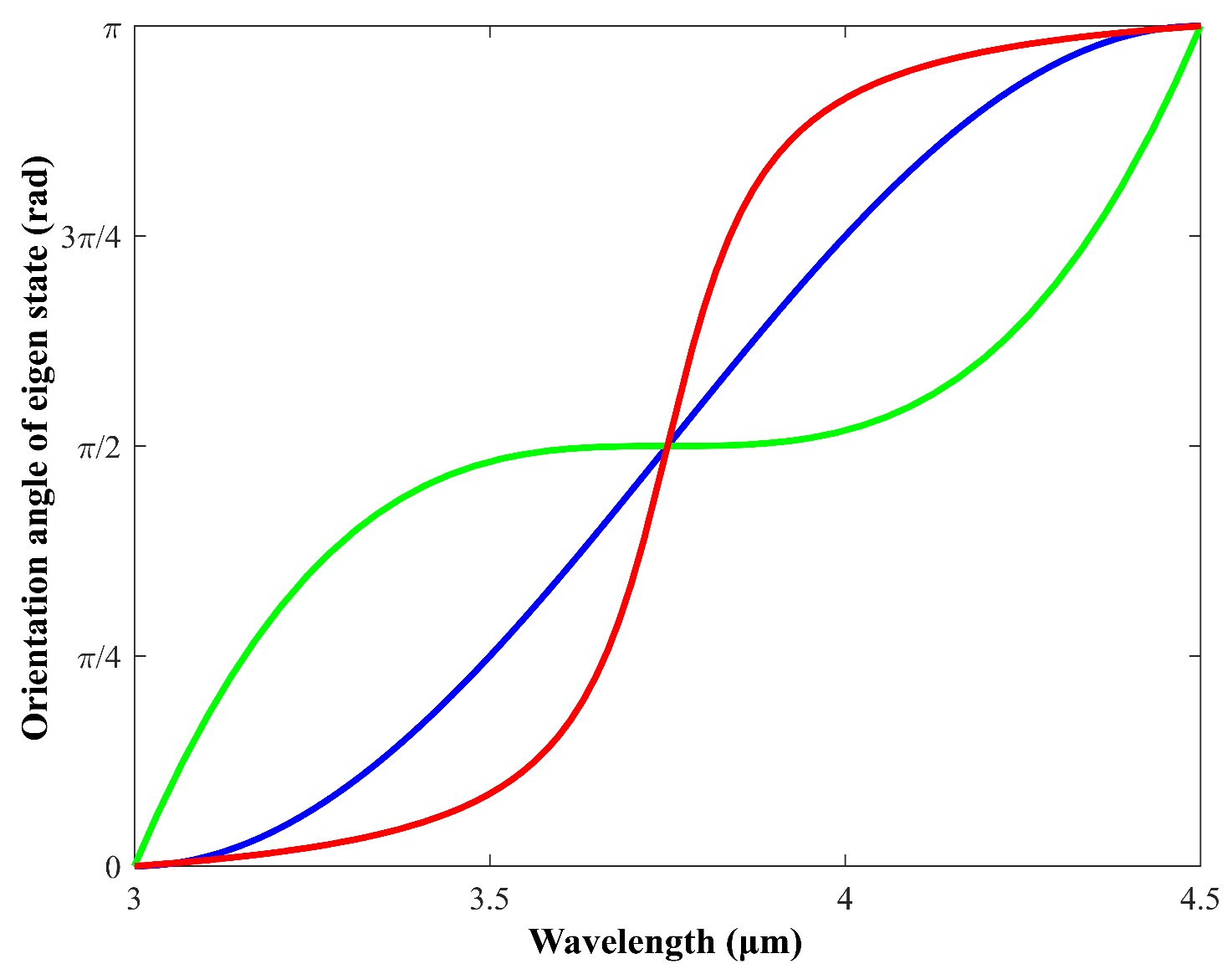


**Fig. s3** Equivalent orientation angle plots as a function of wavelength for demonstration. The three curves represent three different supercell from optimization.

Supplementary Note 3 Optimization with the evolution algorithm

With the dispersive Jones matrix method, different orthogonal polarization states can be realized simultaneously. However, since there are 12 degrees of freedom that need to be optimized, the calculating amount dramatically increases. In our case, each degree of freedom has at least 100 data points and a minimum of 10^24^ optimized data spaces needs to be generated. It is much intractable for traditional optimization algorithms in this case. To save time and ensure the accuracy, we write an updated evolution algorithm through combining the particle swarm optimization (PSO) algorithm and the genetic algorithm (GA). The evolution algorithm is also called random algorithm, which is aimed to improve the speed but sacrifice the accuracy. The PSO algorithm and GA algorithm are widespread in the evolution algorithm family, but their shortcomings are also obvious. The PSO algorithm has a faster convergence speed than the GA algorithm but has lower accuracy. Therefore, we combine the two algorithms, particularly, the PSO algorithm can improve the early convergence speed and the GA algorithm can improve the accuracy of the final stage of optimization. Besides, we also introduce the death rate to accelerate the convergence process. The flow chart is shown in **Fig. s4**.


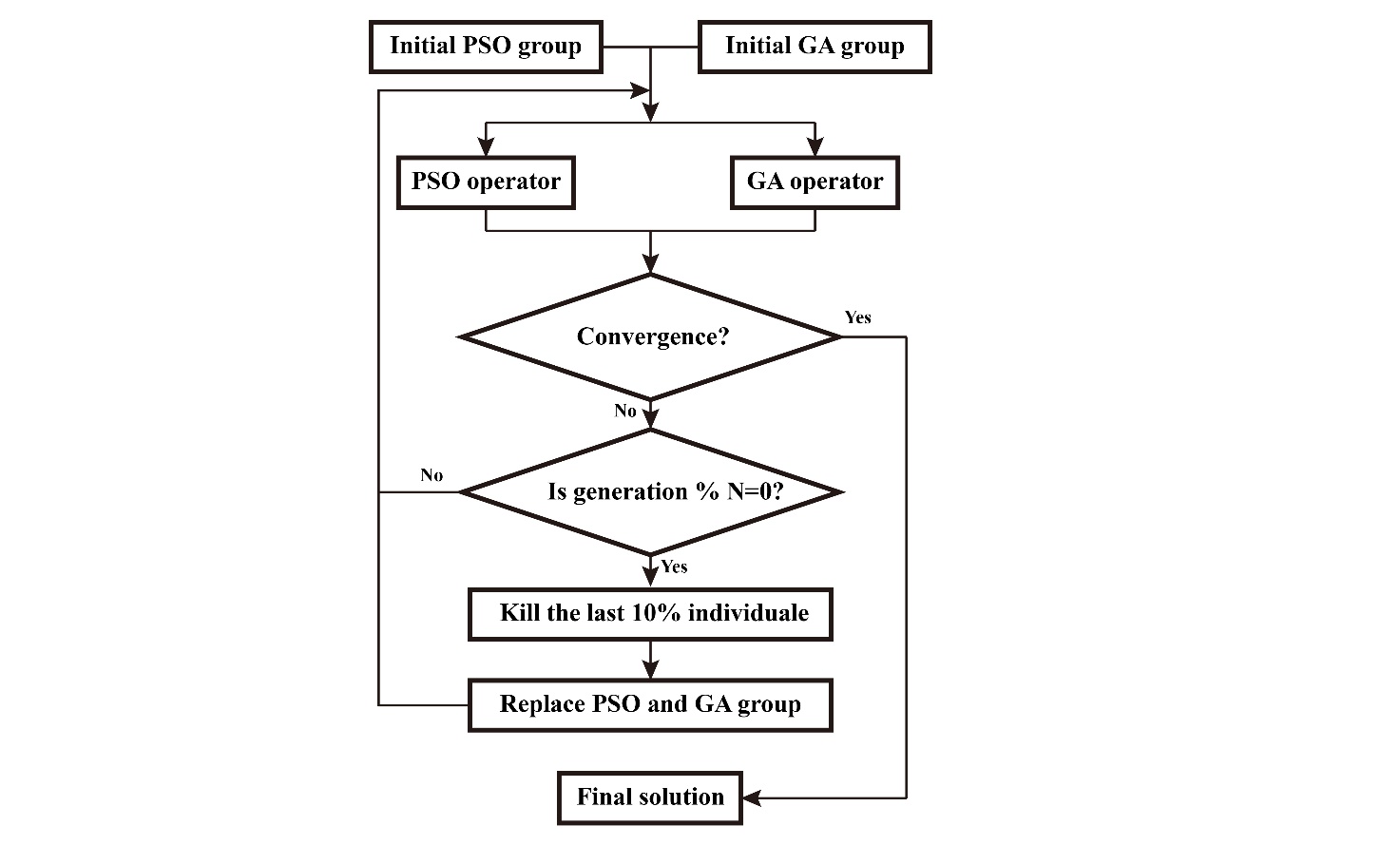


**Fig. s4** Flow chart of the hybrid evolutionary algorithm. The combination of PSO and GA algorithms accelerates the convergence and introduces the death rate to jump out of the local optimal value.

Supplementary Note 4

**Table s2** illustrates the comparison of the reported works [s1-s5] with this work in aspects like the numbers of operation wavelengths, requirements on the polarization, the numbers of orthogonal polarization states et al.

**Table s2** Comparison with reported works.

|  | Operation Wavelength | Polarization State Form | Orthogonal Polarization Pair | Polarization Channel | Segmented/interleaved |
| --- | --- | --- | --- | --- | --- |
| **Refs. s1-4** | **One** | **Linear/**  **Circular** | **One** | **One/Two** | **No** |
| **Ref. s5** | **One** | **Arbitrary** | **One** | **Two** | **No** |
| **Ref. s6** | **Three** | **Circular** | **One** | **Two** | **No** |
| **Ref. s7** | **Three** | **Linear** | **One** | **Three** | **No** |
| **Ref. s8** | **One** | **Linear** | **One** | **Three** | **No** |
| **Ref. s9** | **Three** | **Arbitrary** | **Two** | **One** | **Yes** |
| **This work** | **Unlimited*** | **Arbitrary** | **Unlimited*** | **Unlimited*** | **No** |

*The operation wavelength number and related orthogonal polarization pairs’ number are unlimited in theory.

**Fig. s5** illustrates examples of trichromatic and pentachromic focusing under different pairs of orthogonal polarizations. It should be noted that the metaatoms are selected from the database built in this work.


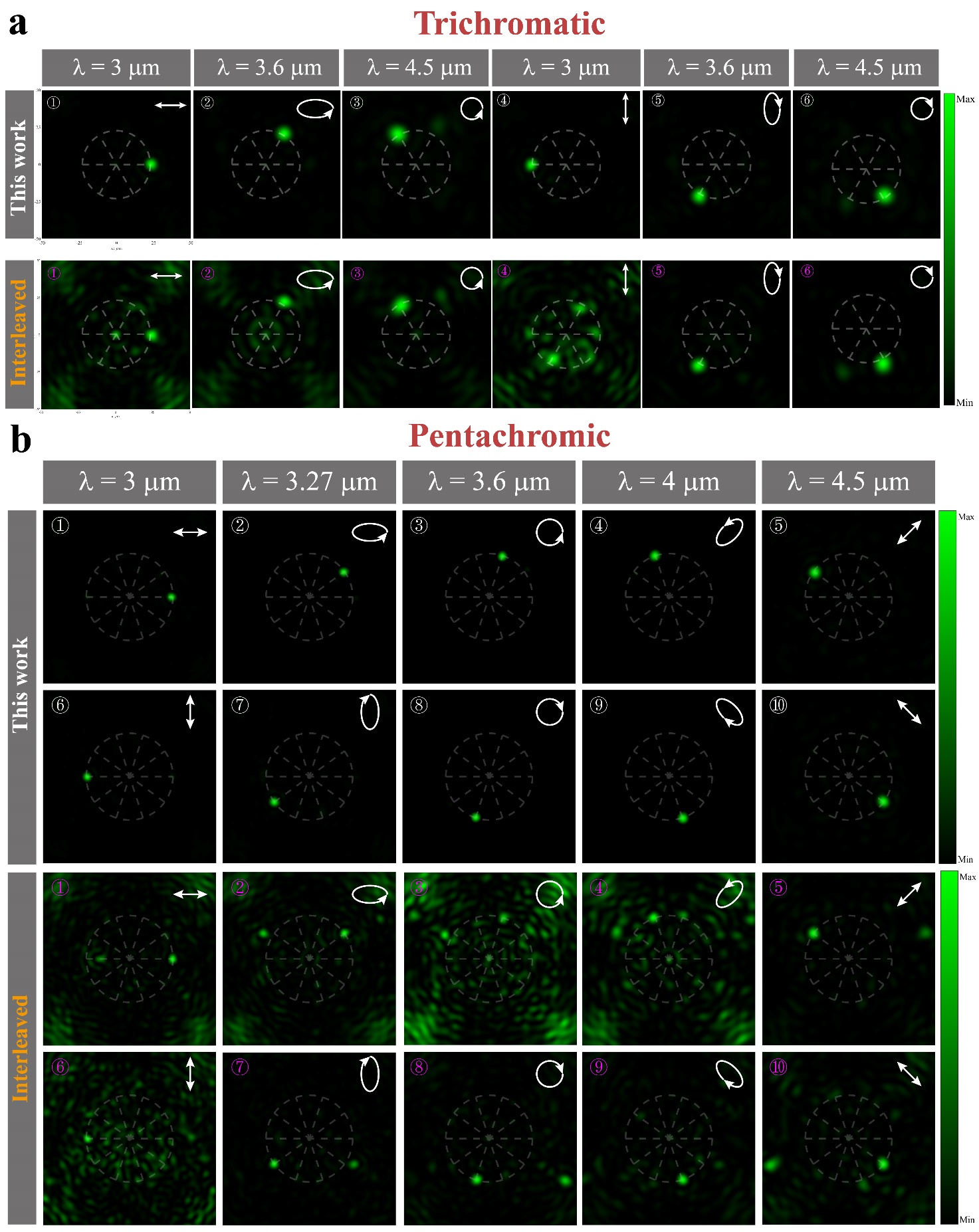


**Fig. s5** Comparison of **a** trichromatic focusing under six polarization states and **b** pentachromic focusing under ten polarization states with method in this work and interleaved method.

Supplementary Note 5 Measurement setup

We built up a measurement system to characterize the metadevice. As shown in **Fig. s6**, blackbody which is a broadband thermal radiator, is adopted as the light source. The linear polarizer and liquid crystal retarder (LCC1113-MIR) are used to modulate the polarizations of incident light. In the light path, the sample is vertically fixed on the hollow acrylic sample rack. It can be finely adjusted for alignment and focus through tuning the six-axis translation and rotation stage. The microscopic module is composed of a 4-mm aspheric lens and a 25-mm lens to magnify the focal spots. The transmitted light after interacting with the metadevice is then captured by the mid-wave infrared camera which is cooled at around 80 K with Stirling cryocooler.


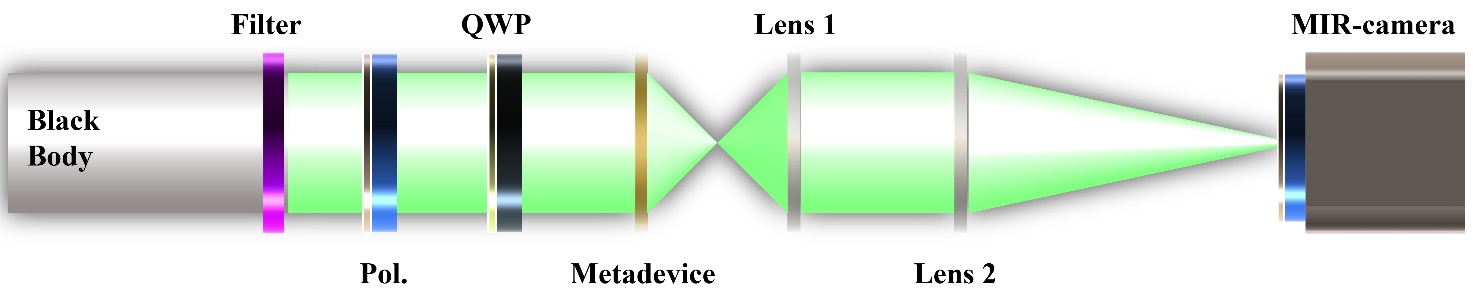


**Fig. s6** Measurement setup. The measurement system is composed with a blackbody operation at 800 K as a broadband light source, filters, polarizers (Pol.), a quarter-wave plate (QWP), and a microscope module. It is used to observe and adjust the sample. A mid-infrared camera cooled at 80K is used to capture the magnified images after interactions with metadevices.

Supplementary Note 6 Relevant results of the polychromatic metadevice

With the above optimizations, the required geometric dimensions are obtained at each position (*x*, *y*). The metadevice with diameter of 100 μm (the same numerical aperture as that in the context) was simulated with the FDTD method. The simulated intensity distributions of the focal spots at each polarization state are showed in **Fig. s7a**. **Fig. s7b** shows the intensity profiles on the lines across the focal spots, from which the full widths at half maximum (FWHMs) are also depicted.


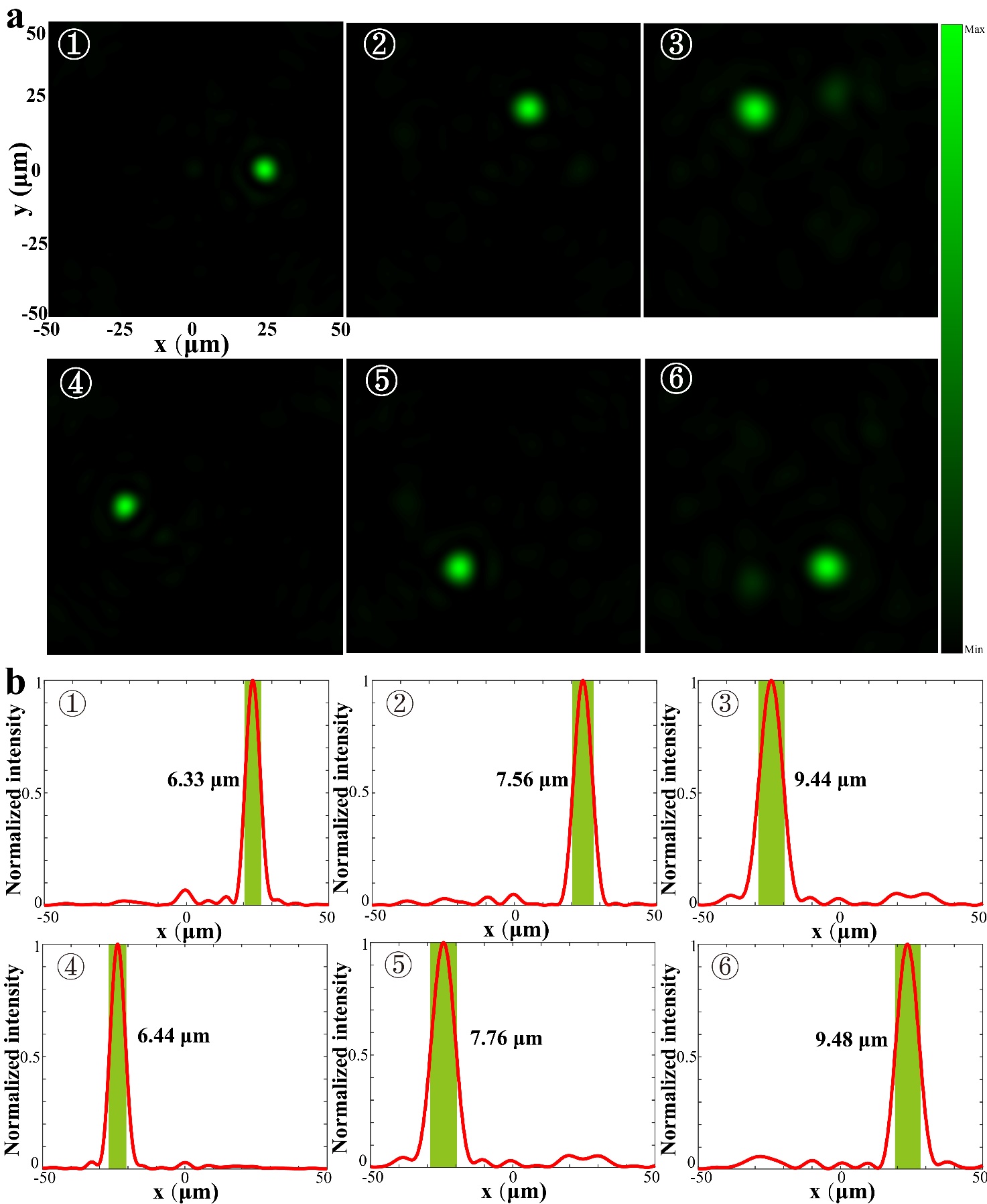


**Fig. s7** **a** Simulated focal spots of metadevice which correspond to the 3 μm *x*-polarization (No. 1), 3.6 μm left-handed elliptical polarization (No. 2) (the long axis is on the *x*-axis), 4.5 μm left-handed circle polarization (No. 3), 3 μm *y*-polarization (No. 4), 3.6 μm right-handed elliptical polarization (No. 5) (the long axis is on the y-axis) and 4.5 μm right-handed circular polarization (No. 6) respectively. **b** Normalized intensity profiles of the lines across the focal spots.

As theoretical predications, six focal spots on different polarization states are separated in the cross-section plane as shown in **Fig. s8**. The focal length keeps almost unchanged with different wavelengths. With a larger device size, the intensity contrast between independent channels is much more obvious.


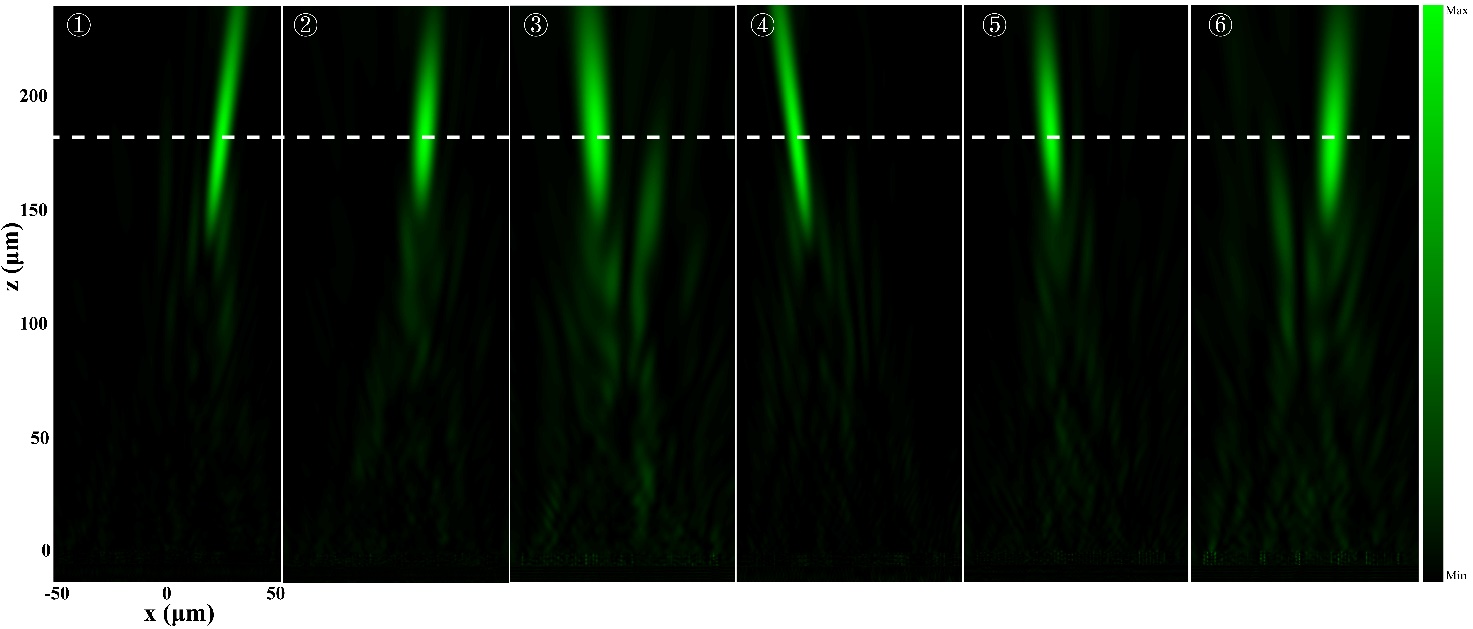


**Fig. s8** Simulated intensity profiles along the axial plane for each polarization at designed wavelengths as indicated Nos. 1-6. The white dashed lines indicate the positions of the mean focal lengths for all the polarization states.

To clearly illustrate the crosstalk, we showed the 3D contour plot of 3-wavelength and 5-wavelength full-polarizations metalens in **Figs. s9** and **s10**.


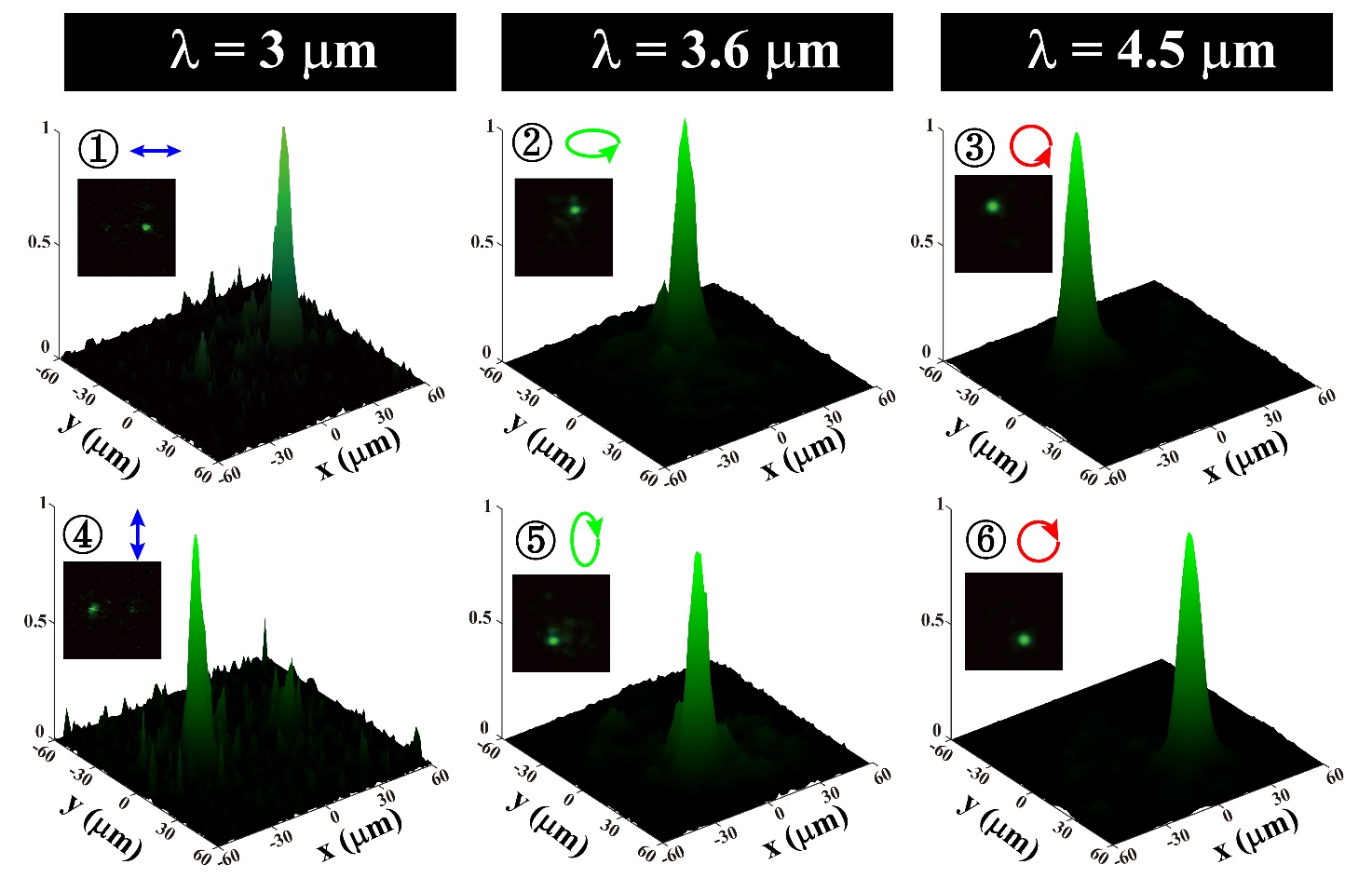


**Fig. s9** Measured 3D contour plot of three-wavelength channel full-polarization metalens.


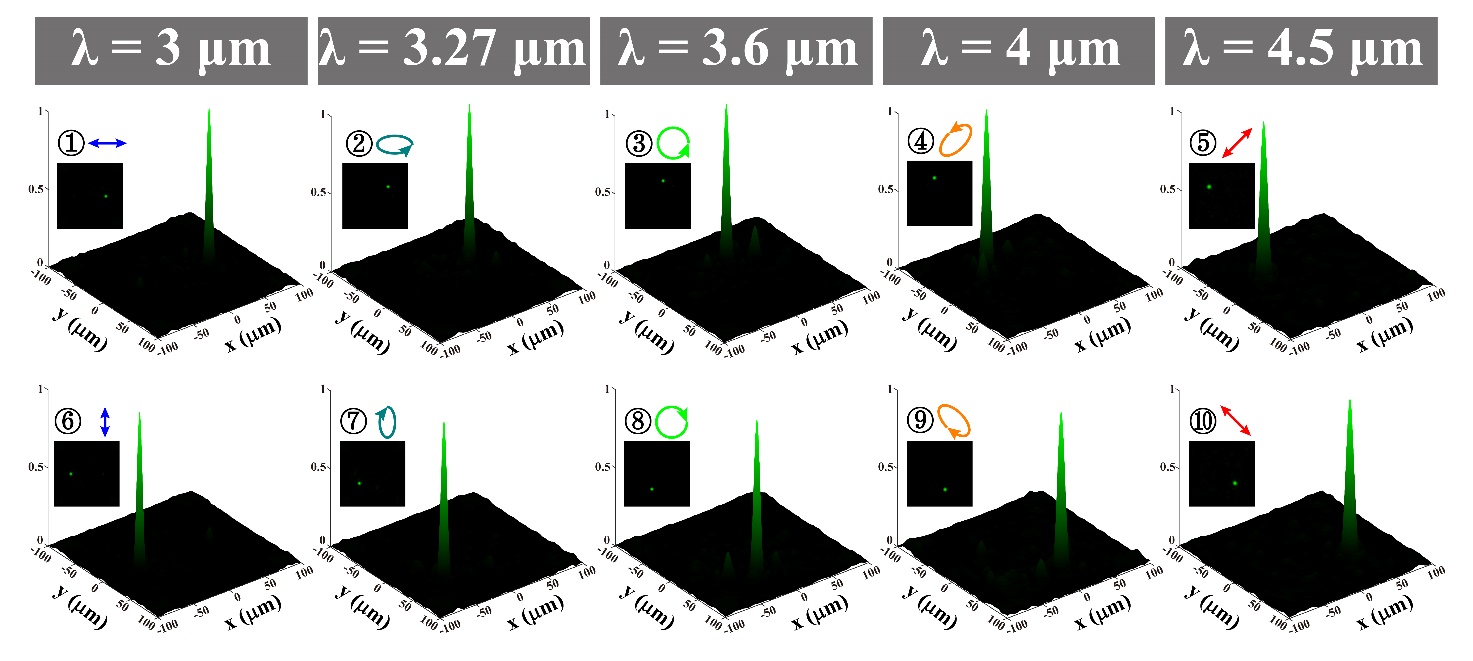


**Fig. s10** Simulated 3D contour plot of five-wavelength channel full-polarization metalens.

Though the metadevices are elaborately optimized to operate at single wavelength, in real cases the devices have bandwidths. Therefore, for a given wavelength range, the upper operation wavelength channel number is limited by the bandwidth. To illustrate this, we calculate the bandwidths for the metadevice with size 200×200 μm^2^ and focal length 400 μm, and the results is showed in **Fig. s11**. The operating bandwidths are 270 nm, 390 nm and 660 nm at three wavelengths respectively.


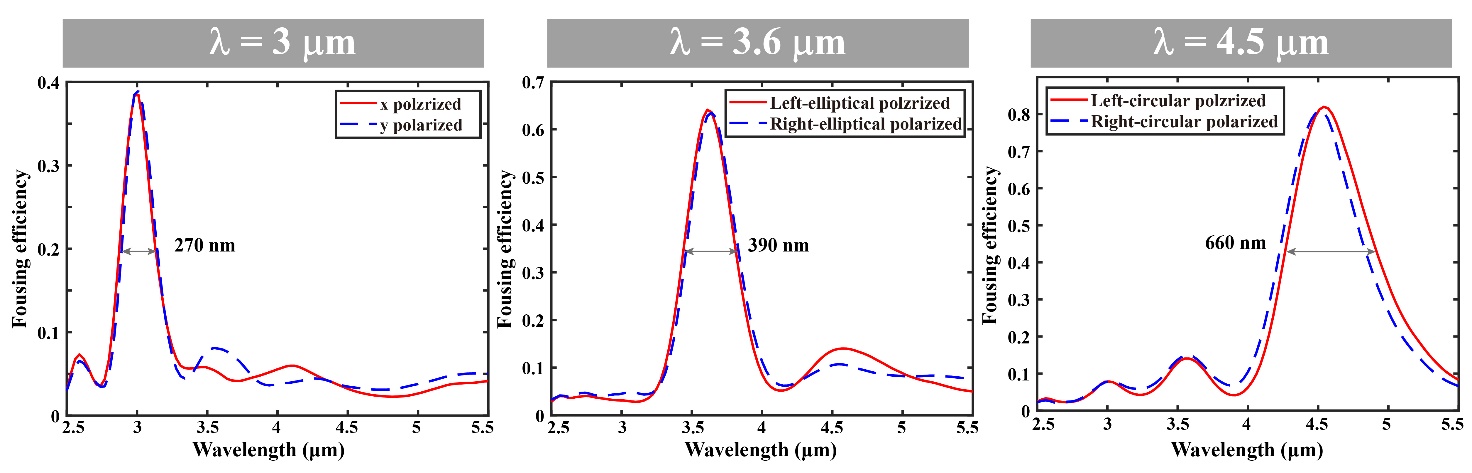


**Fig. s11** The focusing efficiency varies with the wavelength of the six polarization channels. The metadevice size used in the simulation is 200 μm×200 μm. The focal length is 400 μm.

Supplementary Note 7 Realization of polychromatic polarization optical vortex metadevice

Based on the dispersive Jones matrix method, we further design a more complicated metadevice with different topological charges on each channel. Six spots on the focal plane represent the 3 μm *x-p* with topological charge number *l* = 2 (No. 1), 3.6 μm LEP with *l* = 3 (No. 2), 4.5 μm LCP with *l* = 4 (No. 3), 3 μm *y-p* with *l* = -2 (No. 4), 3.6 μm REP with *l* = -3 (No. 5), and 4.5 μm RCP with *l* = -4 (No. 6). The optimization values are shown in **Fig. s12a**. **Fig. s12b** shows the simulated intensity distributions of the metadevice. It can be seen that the position and diameter of the optical vortex singularity are in consistent with the design. Due to the limitation of the computing capacity, smaller size with the same numerical aperture is adopted in the simulation, leading to the degradation of the vortex generation.


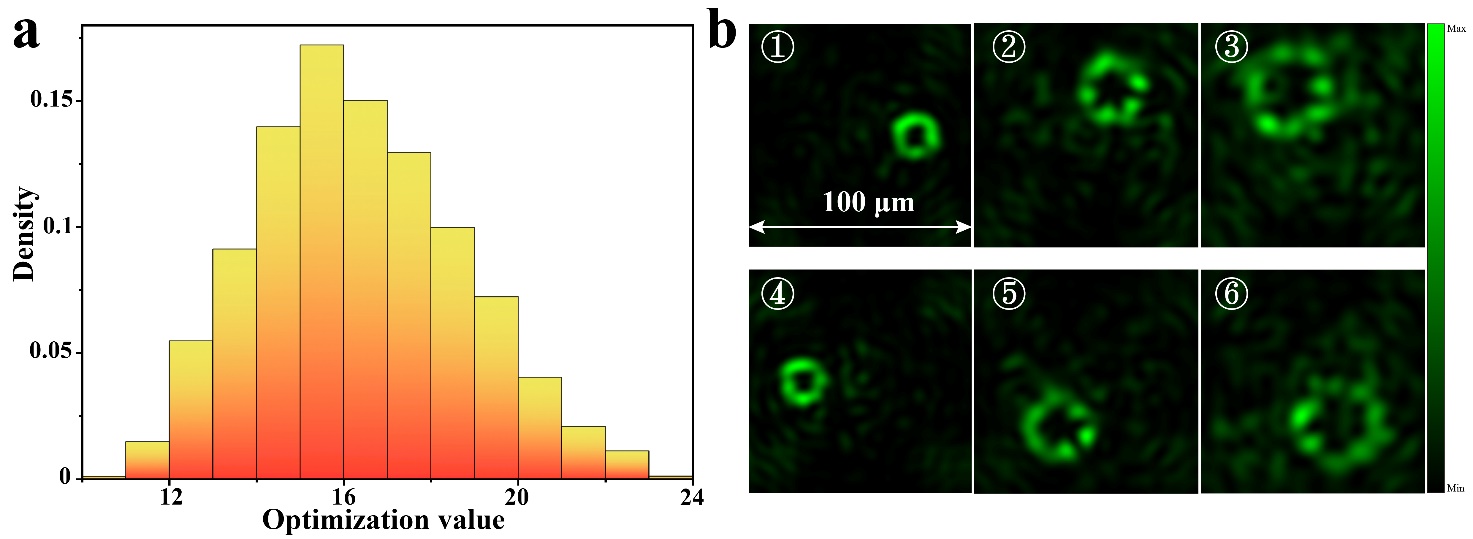


**Fig. s12** **a** Merit function values of the designed metadevice for the generation of vortex beams. The maximum value is 24. **b** Simulated focal spots with different topological charges on the focal plane.

Supplementary Note 8 Subwavelength gratings design

The image patterns are composed of three anisotropic reflective subwavelength gratings manufactured in a bare silicon wafer. The periods of the three gratings are 1.8 μm, 3.3 μm and 3.4 μm, and the widths are 1.3 μm, 0.6 μm, 0.8 μm. The Grating 3 is along the *x*-direction and the other two gratings, i.e., Gratings 1 and 2 are along the *y*-direction. The vertical gray zones in **Figs. s13a-b** represent the working bandwidths and corresponding polarization states, i.e., 3 μm *x-p*, 3.6 μm *y-p*, and 4.5 μm *x-p*. It can be seen that the subwavelength gratings have large polarization isolations at designed wavelengths. **Fig. s13c** shows the SEM image of fabricated samples. The optimization values are shown in **Fig. s13d**.


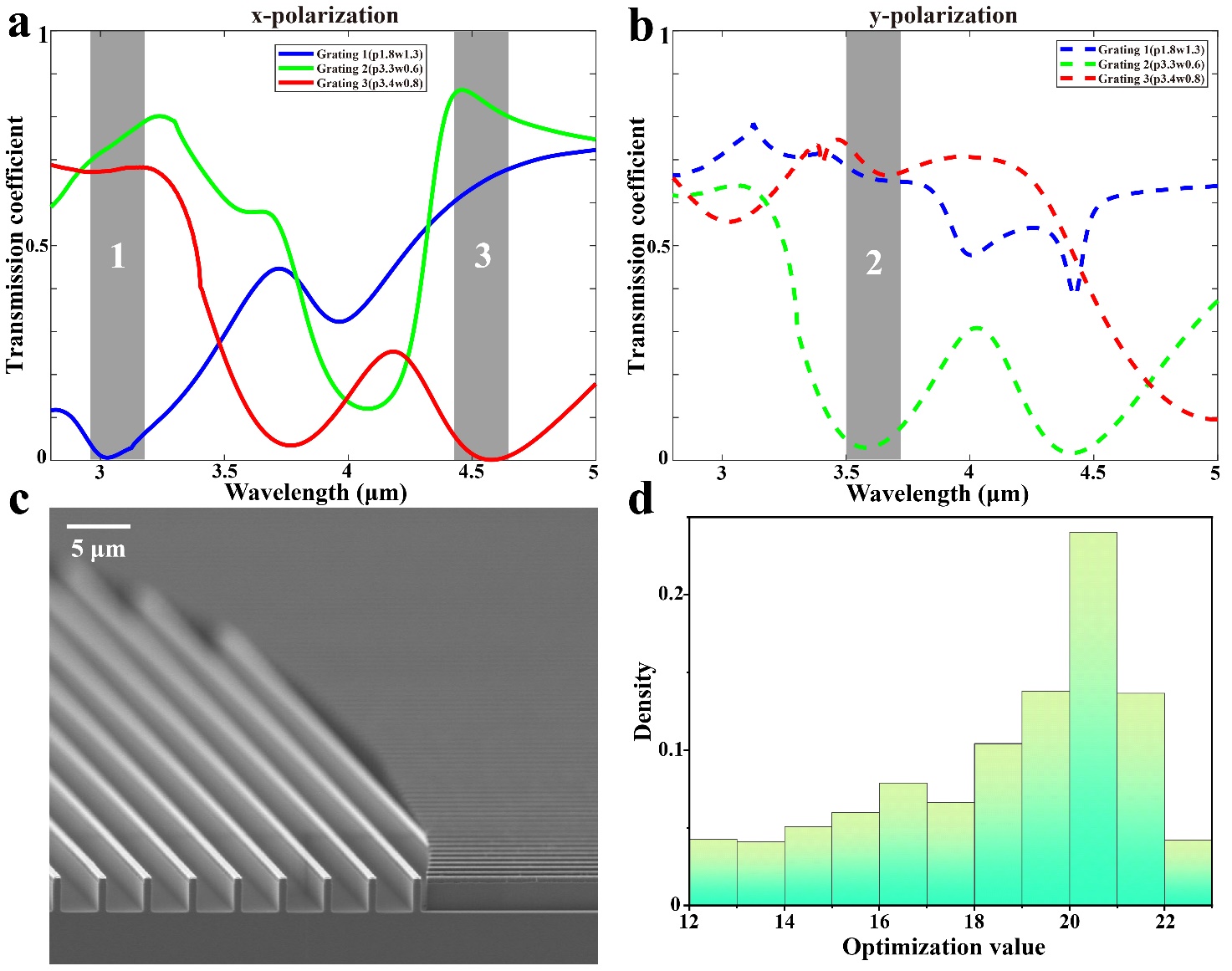


**Fig. s13** **a & b** Transmission spectra of the designed gratings under different polarizations. The operating bandwidth of the imaging metadevice is highlighted with the gray color. **c** SEM image of the fabricated sample with a thickness of 2 μm. **d** Merit values of the designed metadevice.

Supplementary Note 9 Metadevice design for imaging and its characterization of impellers

To characterize the imaging patterns constructed of three gratings above, we fabricate a polarization-selective metalens in a diameter of 1 mm as shown in **Fig. s14a**. The corresponding focusing performance under different polarization states at the designed wavelengths are also illustrated. In addition to the panda pattern, we also fabricate a series of multiple impellers with the subwavelength gratings. **Figs. s14b-d** show the experimental results with the designed metalens. Those results further validate the flexibility of the method.


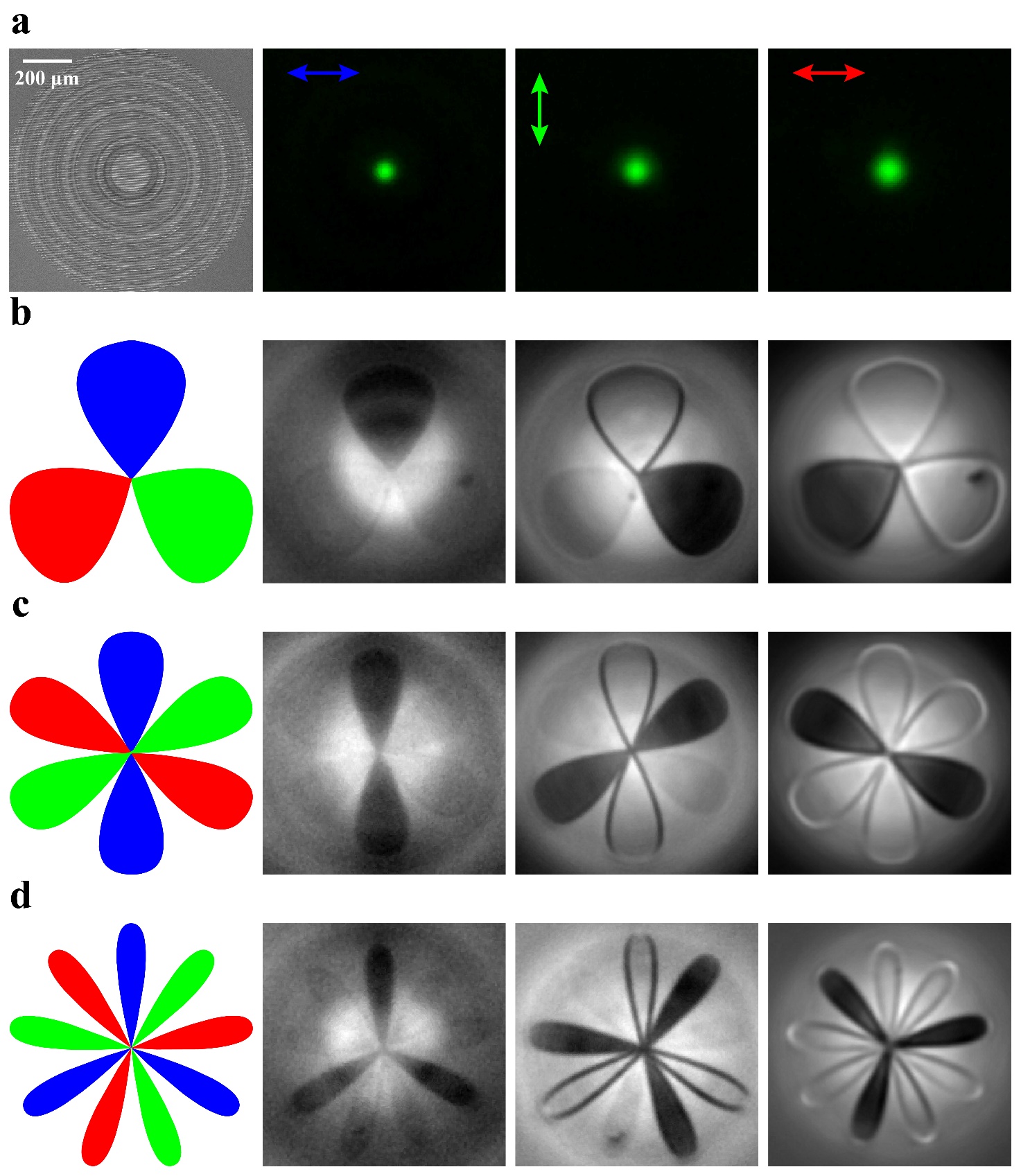


**Fig. s14 a** SEM image of the fabricated metadevice and the focusing performance at different polarizations and wavelengths. **b-d** Schematics of different impellers and the corresponding imaging results with the designed metalens.

Supplementary Note 10 Three-wavelength channel full-polarization holograms

To reflect the capability of generating holograms with our method, we numerically simulated six independent holograms under three orthogonal polarization pairs at three wavelengths, where the polarization states and wavelengths are the same as that in main text. The results show that complex patterns are generated with good contrast to the background. It’s worth noting that the performance can be enhanced with larger simulation area and more supercells. Limited by the calculation memory, the whole simulation size is 300×300 μm^2^ in creating the holograms. The three-wavelength 6-channel holograms of the mid-infrared are shown in **Fig. s15**.


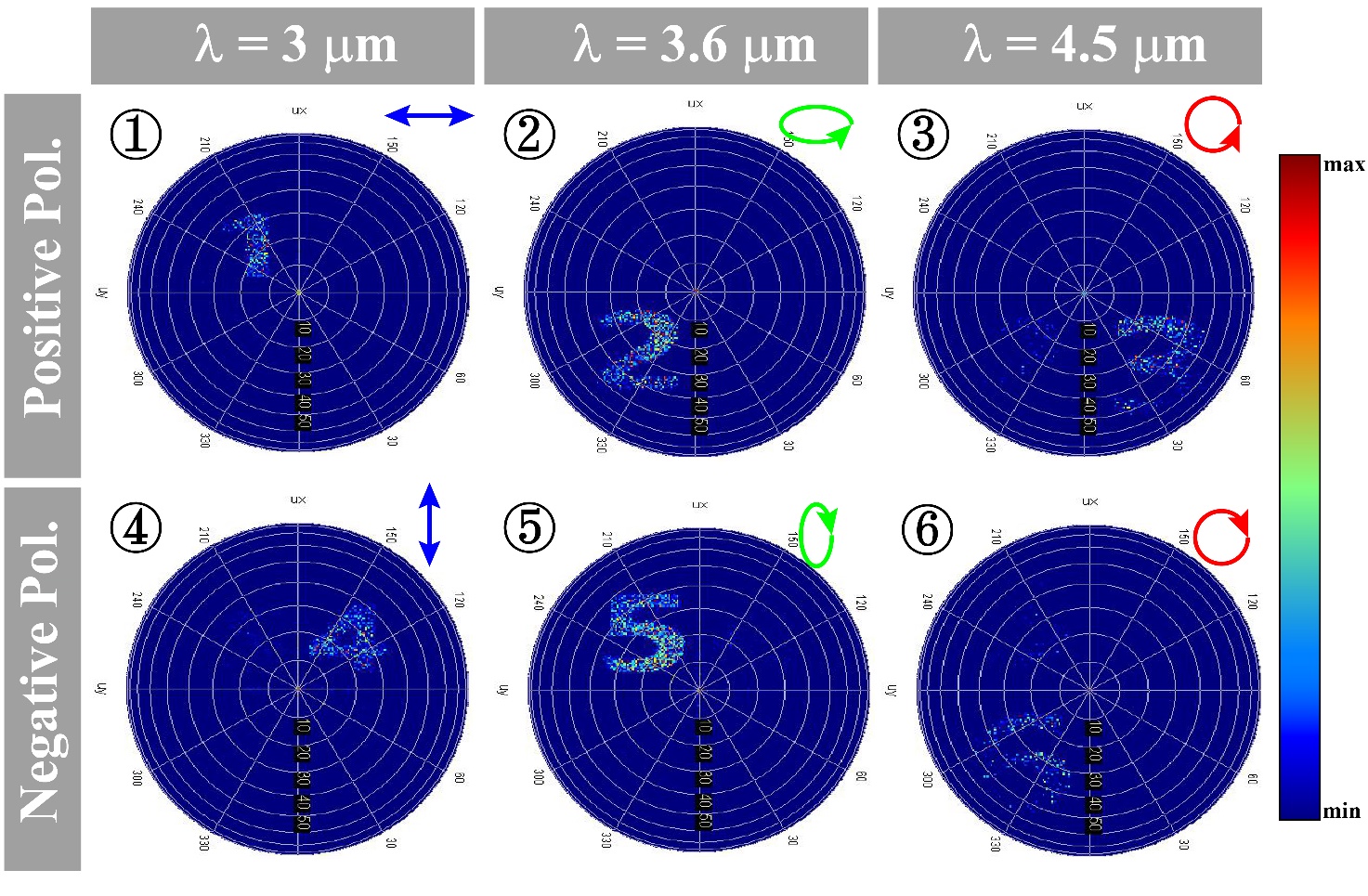


**Fig. s15** The six independent holograms with arbitrary orthogonal polarization pairs located at three wavelengths. The imaging performance can be enhanced with larger simulation area and more supercells. Limited by the calculation memory, the whole simulation size is 300×300 μm^2^ in the simulation.

Reference

s1. Yu N*, et al.* Light propagation with phase discontinuities: Generalized laws of reflection and refraction. *Science* **334**, 333-337 (2011).

s2. Lin J*, et al.* Polarization-controlled tunable directional coupling of surface plasmon polaritons. *Science* **340**, 331-334 (2013).

s3. Mohammadreza Khorasaninejad WTC, Robert C. Devlin, Jaewon Oh, Alexander Y. Zhu, Federico Capasso. Metalenses at visible wavelengths: Diffraction-limited focusing and subwavelength resolution imaging. *Science* **352**, 1190-1194 (2016).

s4. Song Q, Odeh M, Zuniga-Perez J, Kante B, Genevet P. Plasmonic topological metasurface by encircling an exceptional point. *Science* **373**, 1133-1137 (2021).

s5. Balthasar Mueller JP, Rubin NA, Devlin RC, Groever B, Capasso F. Metasurface polarization optics: Independent phase control of arbitrary orthogonal states of polarization. *Physical Review Letters* **118**, 113901 (2017).

s6. Jin L*, et al.* Noninterleaved metasurface for (2(6)-1) spin- and wavelength-encoded holograms. *Nano Letters* **18**, 8016-8024 (2018).

s7. Hu Y*, et al.* Trichromatic and tripolarization-channel holography with noninterleaved dielectric metasurface. *Nano Letters* **20**, 994-1002 (2020).

s8. Bao Y, Wen L, Chen Q, Qiu C-W, Li B. Toward the capacity limit of 2D planar Jones matrix with a single-layer metasurface. *Science Advances* **7**, eabh0365 (2021).

s9. Guo X*, et al.* Full‐color holographic display and encryption with full‐polarization degree of freedom. *Advanced Materials* **34**, 2103192 (2021).
